# Interpretable machine learning models for predicting skip metastasis in cN0 papillary thyroid cancer based on clinicopathological and elastography radiomics features

**DOI:** 10.3389/fonc.2024.1457660

**Published:** 2025-01-07

**Authors:** Xiaohua Yao, Mingming Tang, Min Lu, Jie Zhou, Debin Yang

**Affiliations:** ^1^ Departments of Ultrasound, Jiading District Central Hospital Affiliated Shanghai University of Medicine &Health Sciences, Shanghai, China; ^2^ Department of Endocrinology, Jiading District Central Hospital Affiliated Shanghai University of Medicine & Health Sciences, Shanghai, China

**Keywords:** papillary thyroid cancer, machine learning, clinically node-negative (cN0), skip lymph node metastasis, radiomics

## Abstract

**Background:**

Skip lymph node metastasis (SLNM) in papillary thyroid cancer (PTC) involves cancer cells bypassing central nodes to directly metastasize to lateral nodes, often undetected by standard preoperative ultrasonography. Although multiple models exist to identify SLNM, they are inadequate for clinically node-negative (cN0) patients, resulting in underestimated metastatic risks and compromised treatment effectiveness. Our study aims to develop and validate a machine learning (ML) model that combines elastography radiomics with clinicopathological data to predict pre-surgical SLNM risk in cN0 PTC patients with increased risk of lymph node metastasis (LNM), improving their treatment strategies.

**Methods:**

Our study conducted a retrospective analysis of 485 newly diagnosed primary PTC patients, divided into training and external validation cohorts. Patients were categorized into SLNM and non-SLNM groups based on follow-up outcomes and postoperative pathology. We collected preoperative clinicopathological data and extracted, standardized radiomics features from elastography imaging to develop various ML models. These models were internally validated using radiomics and clinicopathological data, with the optimal model’s feature importance analyzed through the Shapley Additive Explanations (SHAP) approach and subsequently externally validated.

**Results:**

In our study of 485 patients, 67 (13.8%) exhibited SLNM. The extreme gradient boosting (XGBoost) model, integrating elastography radiomics with clinicopathological data, demonstrated superior performance in both internal and external validations. SHAP analysis identified five key determinants of SLNM: three radiomics features from elastography images, one clinical variable, and one pathological variable.

**Conclusion:**

Our evaluation highlights the XGBoost model, which integrates elastography radiomics and clinicopathological data, as the most effective ML approach for the prediction of SLNM in cN0 PTC patients with increased risk of LNM. This innovative model significantly enhances the accuracy of risk assessments for SLNM, enabling personalized treatments that could reduce postoperative metastases in these patients.

## Introduction

Lymph node metastasis (LNM) significantly influences surgical approaches and recurrence risk stratification in papillary thyroid cancer (PTC) (1, 2). PTC cells spread through the lymphatic system in a sequential manner, initially involving the central compartment, then progressing to the ipsilateral lateral compartment, and ultimately metastasizing to the contralateral, lateral or mediastinal compartments (3). Skip lymph node metastasis (SLNM) is a rare phenomenon where cancer bypasses the central lymph nodes (CLN) and directly metastasizes to the lateral lymph nodes (LLN) (4). The sensitivity of preoperative ultrasound in detecting CLN is relatively low, with an estimated accuracy of only 30–55% (5, 6). Moreover, ultrasound often fails to identify abnormal lymph nodes smaller than 5 mm in diameter (7). Despite undergoing central lymph node dissection (CLND), there may be an insufficient number of CLN sampled. Due to these limitations, patients may still be inaccurately diagnosed as clinically node-negative (cN0), a false-negative diagnosis, despite the presence of SLNM. This underestimation of their metastatic risk can impact treatment strategies. Prophylactic lateral neck dissection is not the standard treatment for PTC patients with cN0 status (2). While mortality for patients who experience lymph node recurrence remains low, it is indeed the case that LNM after surgery might necessitate additional interventions, including further surgery (8–10) and selectively applied radiotherapy (11, 12). Several models have been developed to distinguish patients with SLNM from those with typical LNM (13–15). However, these models have limited clinical applicability as they are only suitable for evaluating the likelihood of SLNM in clinically node-positive (cN+) PTC, not in cN0 cases. Studies indicate that the surgical and treatment approaches for cN+ PTC patients remain the same, regardless of the presence of SLNM. While accurately identifying SLNM in cN0 PTC patients could facilitate more personalized treatment strategies, like prophylactic lymph node dissection, existing models struggle to predict SLNM reliably in the preoperative setting.

Previous research has linked clinicopathological factors like age, tumor location and Ki-67 to LNM in PTC (13–16). Although these indicators are useful, they do not encompass all the predictive data available from patients. Recent research underscores that fine needle aspiration with thyroglobulin (FNA-Tg) measurement from eluates is a reliable method for detecting cervical LNM, demonstrating significant diagnostic accuracy (17, 18). However, it is important to note that the optimal cutoff value for FNA-Tg remains a subject of debate, with suggested values ranging widely from 0.2 to 36 ng/mL, and the method is an invasive (19). The standardization of the FNA-Tg procedure has yet to be achieved. While ultrasound is an accessible, cost-effective, and non-invasive diagnostic tool, its sensitivity varies significantly across different anatomical compartments-approximately 62–94% for the lateral compartment and 30–55% for the central compartment (5, 6), and its effectiveness is limited for micrometastases (7). Elastography, a novel ultrasound-based technique, assesses tissue elasticity primarily for the non-invasive assessment of lesions. It enhances conventional ultrasound examinations by introducing stiffness as an additional measurable property (20, 21). However, elastography parameters lack high-dimensional characteristics across various frequency scales. Radiomics, a method that extracts medical image features through high-throughput techniques, provides a quantitative and objective basis for standardized analysis (22). This approach has recently been applied to ultrasound images of fibrosis (23–25). However, the relationships between the diverse and detailed features identified by radiomics and their clinical outcomes are intricate and often nonlinear, presenting significant analytical challenges. This complexity renders traditional linear predictive models, like logistic regression (Logit), less effective in achieving precise predictions. Consequently, the use of machine learning (ML), a branch of artificial intelligence known for its ability to decipher complex patterns in large data sets, is crucial for developing effective predictive models (26). Common ML classifiers, including support vector machine (SVM) and extreme gradient boosting (XGBoost), have shown versatility in predicting the progression of conditions like liver disease, hypertensive intracerebral hemorrhage, and breast cancer (27–29). Yet, research remains limited on ML models that leverage elastography radiomics to predict SLNM preoperatively in cN0 PTC patients.

With this background, our study seeks to develop and validate an interpretable ML model that utilizes elastography radiomics features alongside clinicopathological data. The goal is to predict the risk of SLNM in cN0 PTC patients with an increased risk of LNM before surgery, aiming to enhance treatment strategies for these individuals.

## Materials and methods

### Ethics statement

This retrospective study was approved by the ethics committee of Jiading District Central Hospital Affiliated Shanghai University of Medicine &Health Sciences (NO. 2021K07). Informed consent was waived by the Ethics Committee due to the study’s retrospective design. This study was conducted in accordance with the Helsinki Declaration.

### Study population

**Figure 1:**
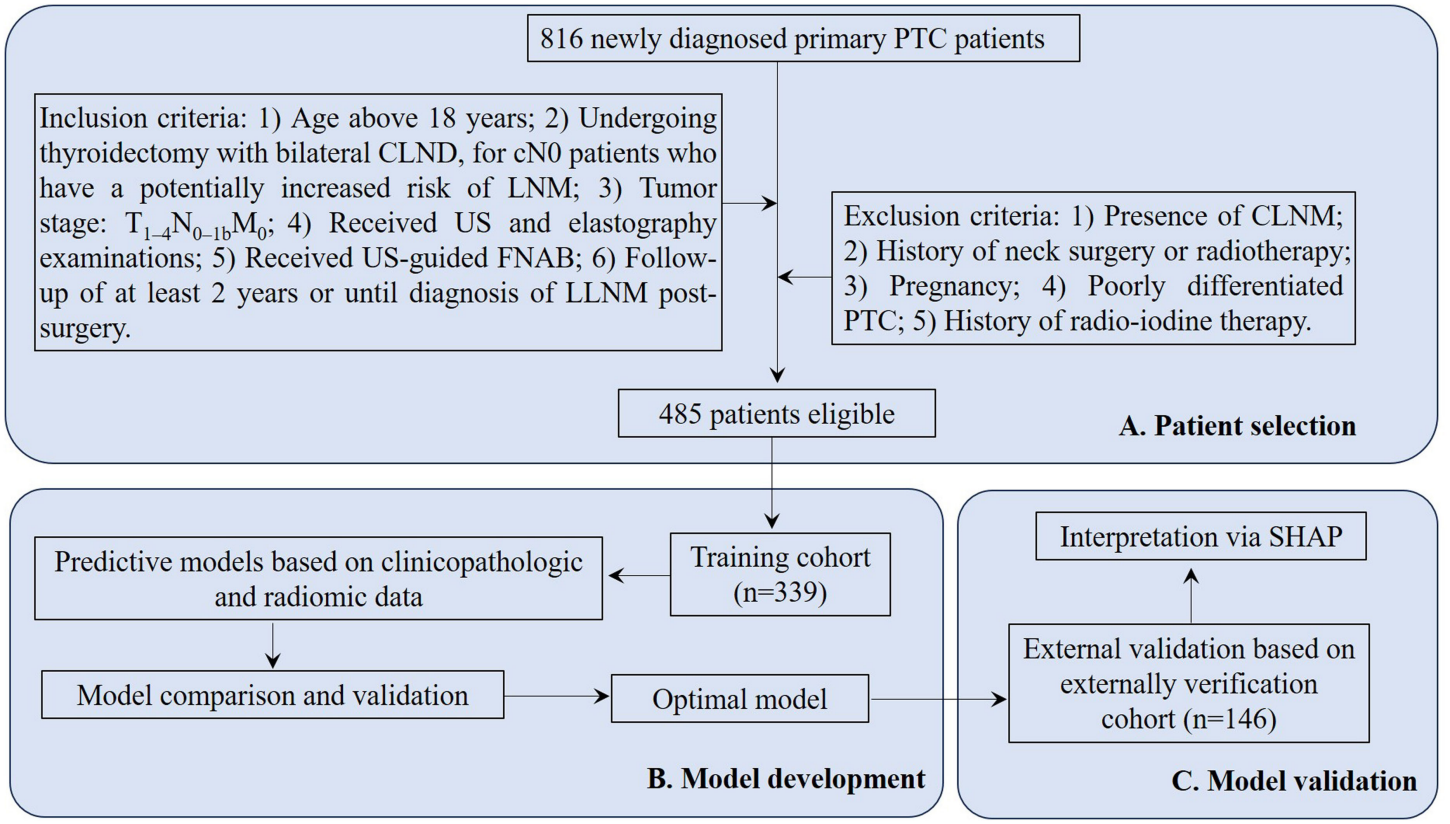
Flowchart for PTC patient selection and cohort distribution for developing and validating predictive model. PTC, papillary thyroid cancer; CLND, central lymph node dissection; cN0, clinically node-negative; LNM, lymph node metastasis; US, ultrasonography; FNAB, fine-needle aspirations biopsy; LLNM, lateral lymph node metastases; CLNM, central lymph node metastasis; SHAP, Shapley Additive Explanations.

This study screened medical records of 816 newly diagnosed primary PTC patients hospitalized at Jiading District Central Hospital Affiliated Shanghai University of Medicine &Health Sciences from January 2017 to December 2021. Inclusion criteria: 1) Age above 18 years; 2) Undergoing thyroidectomy with bilateral CLND, for cN0 patients who have a potentially increased risk of LNM characterized by a tumor diameter >4 cm, multifocal disease, extrathyroidal extension, etc.; 3) Tumor stage: T_1–4_N_0–1b_M_0_; 4) Received US and elastography examinations; 5) Received US-guided fine-needle aspirations biopsy (FNAB); 6) Follow-up of at least 2 years or until diagnosis of LLNM post-surgery. Exclusion criteria: 1) Presence of central lymph node metastasis (CLNM); 2) History of neck surgery or radiotherapy; 3) Pregnancy; 4) Poorly differentiated PTC; 5) History of radio-iodine therapy. Finally, 485 patients were included in this study. Based on the follow-up outcomes and postoperative pathology reports, 67 cN0 patients with postoperative LLNM were assigned to the SLNM group, while 418 without LLNM were categorized as non-SLNM (**Figure 1**).

### Clinical data collection

Preoperative clinical data, including age, gender, and body mass index (BMI), were obtained from the hospital information system.

### US−guided FNAB

Thyroid fine-needle aspirations (FNAs) were conducted under ultrasound guidance by a radiologist. For the procedure, patients were placed in a supine position with elevated backs and tilted heads. A 23-gauge needle (Pajunk, Germany) was used to puncture each thyroid nodule three times. A part of the aspirate was analyzed to assess the expression of Ki-67, P53, and CK-19 using immunocytochemistry. The process involves multiple steps including fixation, embedding, and dehydration. Antibodies used included Ki-67 (dilution 1:50, clone MIB-1), P53 (dilution 1:100, clone DO7), and CK-19 (dilution 1:200, clone RCK108). Stain interpretations were performed by three experienced cytopathologists. For accurate analysis, each case selected contained at least 200 cells to determine the percentage of cells expressing Ki-67, P53, and CK-19. **Supplementary Figure 1** presents a representative report of these markers. FNAB specimens must be evaluated by a skilled cytopathologist and reported following the Bethesda Classification System.

### Acquisition of elastography imaging

B-mode ultrasound and elastography for thyroid nodules were conducted using the Aplio i800 ultrasound system (Canon, Japan). Two sonographers, each with over 10 years of experience in thyroid ultrasound and more than 5 years in elastography, employed a standardized imaging protocol to conduct the sonographic examinations, thereby ensuring consistent elastography image quality. They assessed features like tumor size, distribution, and shape, unaware of the clinicopathologic findings. Elastography was initiated by centering the lesion within the image. After the B-mode ultrasound, the elastography image was captured at the plane displaying the thyroid nodule’s largest diameter.

### Image segmentation and feature extraction

**Figure 2:**
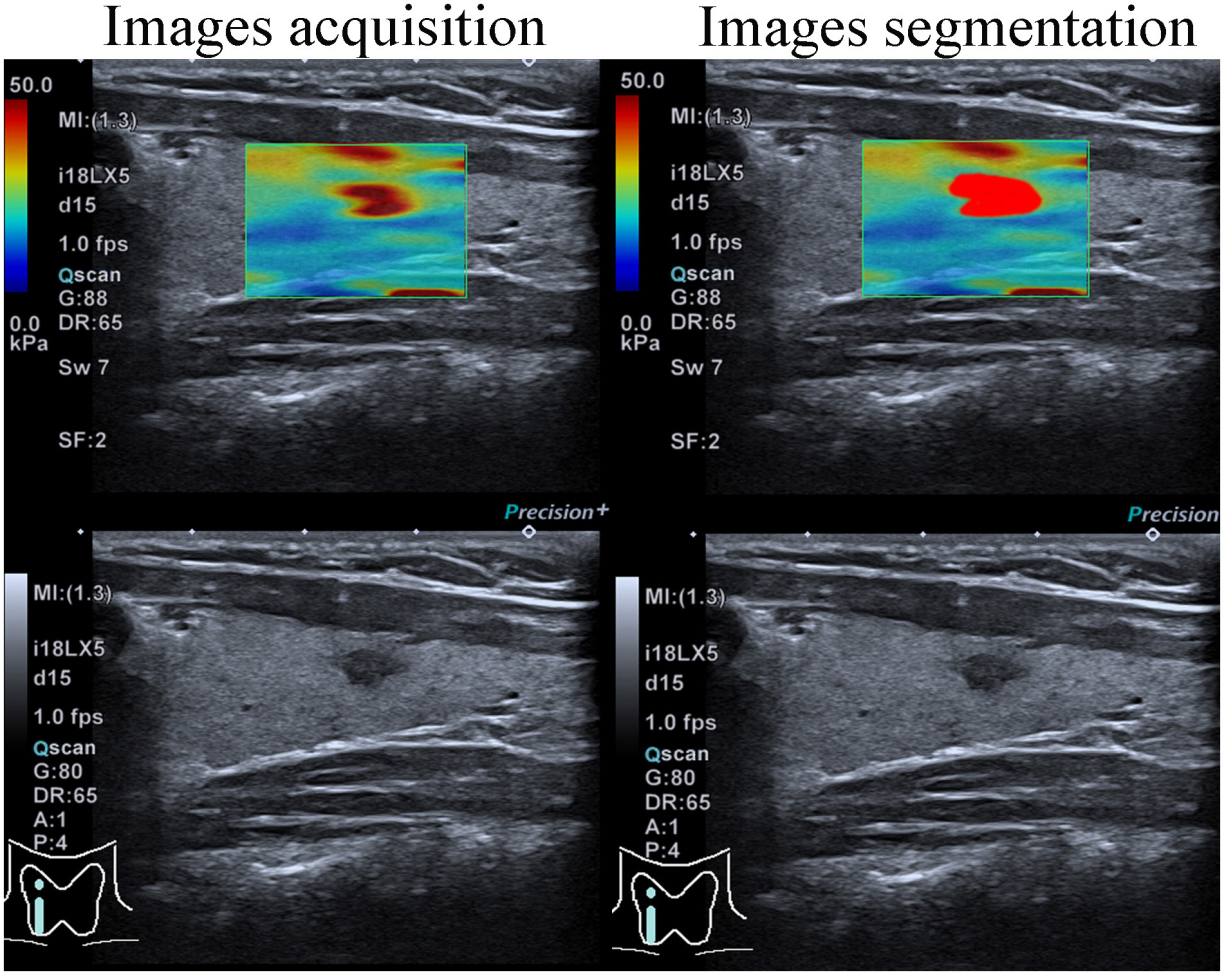
Elastography images were acquired and subsequently segmented.

Elastography thyroid images were processed using segmentation software, 3D Slicer (Version 5.0.2). An experienced ultrasonographer (U1), lacking access to clinical data, delineated the ROI (**Figure 2**). Another ultrasonographer (U2) independently verified these outlines without clinical data, using the same approach. The consistency between their delineations was assessed using intraclass correlation coefficients (ICCs), with values ≥ 0.80 indicating high reproducibility. Radiomic signatures were extracted from each ROI using PyRadiomics (Version 3.7), resulting in 6 image types and 6 feature classes.

### Data preprocessing

Before developing a prediction model, data preprocessing was crucial to eliminate biases. This step standardized all data, including extracted radiomics features and clinicopathological information. Continuous variables were normalized using Z-scores to have a mean of zero and a standard deviation of one, while categorical variables were binarized, assigned values of “0” or “1.”

### Selection of radiomics features

The consistency of feature extraction across different observers was assessed using interclass correlation coefficients (ICCs), with a threshold of 0.80 for acceptable agreement. Then, the Student’s t-test was conducted to identify significant features, considering those with false discovery rate (FDR)-corrected P values below 0.05. Subsequently, a least absolute shrinkage and selection operator (LASSO) logistic regression model was employed to further refine feature selection.

### Derivation and internal validation of ML models

To evaluate the risk of SLNM in cN0 PTC patients, we employed four established ML classifiers: Logit, Random Forest (RF), SVM, and XGBoost. We developed distinct prediction models based on clinicopathological data, radiomics features, and their combinations. During model training, we utilized a triply-repeated five-fold cross-validation to maximize data utilization, dividing the training set into inner training and testing subsets for sequential assessments. For the RF model, we configured it with 500 trees and determined the number of features for node splitting using the square root of the total feature count. The SVM employed a radial basis function (RBF) kernel, effective for non-linear data, with hyperparameters fine-tuned via grid search. The cost parameters were set at [0.1, 1, 10], and gamma parameters for the RBF kernel at [0.001, 0.01, 0.1]. XGBoost was optimized using grid search with parameters including a learning rate of 0.02, a maximum tree depth of 4, and an ensemble of 600 trees, ensuring a balance between model complexity and prediction accuracy, thereby streamlining model development.

After developing each model, we conducted a rigorous internal validation to assess their discrimination, calibration, and clinical applicability. The optimal predictive model was selected based on its superior discriminatory power, robust calibration, and clinical utility.

### Interpretability and external validation of ML models

After identifying the best predictive models, we explored the individual contributions of each variable to the predictions using the SHAP (Shapley Additive Explanations) methodology. This approach enabled a detailed understanding of feature importance, emphasizing the variables with the most significant impact. Features were ranked by their SHAP values in descending order to identify the key predictors in our patient cohort. The SHAP force plot is crafted to analyze and interpret prediction results for individual samples. To ensure the reliability of our models, we conducted external validation. This thorough assessment confirmed their discriminative power, calibration, and clinical relevance, offering a clear view of their predictive strength.

### Statistical analysis

Statistical analyses were conducted using R (Version 4.2.1) and Python (Version 3.7.1). Skewed continuous variables were described as median [interquartile range (IQR)] and assessed using the Mann–Whitney U-test. Categorical variables were presented as number (percentage) and analyzed with the χ^2^ test. Model performance was evaluated through receiver operating characteristic (ROC) curve analysis, specifically focusing on the area under the curve (AUC), and additional metrics like Precision, Recall, and F1 Score to assess discriminative ability. AUC comparisons were made using Delong’s test. Model calibration was evaluated using calibration curves and the Brier Score to measure probability prediction accuracy. For assessing clinical utility, decision curve analysis (DCA) calculated net benefits at various threshold probabilities.

## Results

### Patient characteristics

Data from 816 newly diagnosed primary PTC patients were extracted from the inpatient management system. After rigorous screening according to inclusion and exclusion criteria, 485 patients were selected and split into two cohorts: 339 in the training cohort and 146 in the external verification cohort (**Figure 1**). All enrolled patients were followed regularly for a minimum of two years or until a diagnosis of LLNM post-surgery was confirmed. The follow-up period concluded on December 31, 2023, with a median duration of 51.5 months. Among all patients, 67 (13.8%) were diagnosed with SLNM. The distributions of SLNM are shown in **Table 1**. The prevalence of SLNM was similar in both cohorts—14.5% (49/339) in the training cohort and 12.3% (18/146) in the external verification cohort, with no significant statistical difference (χ^2^ = 0.229, P = 0.632). **Table 2** corroborates these results, showing consistent clinicopathological characteristics across both cohorts without significant differences (all P > 0.05).

**Table 1:** Distribution of skip lymph node metastasis.

Distribution	n=67
Single level, n (%)	
II	11 (16.4)
III	20 (29.9)
IV	7 (10.4)
Two levels, n (%)	
II+III	5 (7.5)
II+IV	4 (6.0)
III+IV	6 (9.0)
III+V	2 (3.0)
IV+V	2 (3.0)
Three levels, n (%)	
II+III+IV	5 (7.5)
III+IV+V	4 (6.0)
Four levels, n (%)	
II+III+IV+V	1 (1.5)

**Table 2:** Comparisons of the clinicopathological characteristics between the training and externally verification cohorts.

Clinicopathological characteristics	Training cohort (N=339)	Externally verification cohort (N=146)	P value
Age, year, median (IQR)	44.00 (35.50, 51.00)	44.00 (36.00, 53.00)	0.232^*^
Gender, n (%)			0.905^#^
Male	101 (29.8)	42 (28.8)	
Female	238 (70.2)	104 (71.2)	
BMI, kg/m^2^, median (IQR)	23.90 (20.90, 27.25)	24.30 (21.85, 27.20)	0.369^*^
Diabetes, n (%)			0.586^#^
Yes	41 (12.1)	21 (14.4)	
No	298 (87.9)	125 (85.6)	
Graves’ disease, n (%)			0.744^#^
Yes	4 (1.2)	3 (2.1)	
No	355 (98.8)	143 (97.9)	
Family history of thyroid cancer, n (%)			0.729^#^
Yes	28 (8.3)	10 (6.8)	
No	311 (91.7)	136 (93.2)	
History of Hashimoto’s thyroiditis, n (%)			0.563^#^
Yes	45 (13.3)	23 (15.8)	
No	294 (86.7)	123 (84.2)	
T stage of tumor, n (%)			0.573^#^
T1	17 (5.0)	6 (4.1)	
T2	192 (56.6)	88 (60.3)	
T3	101 (29.8)	36 (24.7)	
T4	29 (8.6)	16 (11.0)	
Tumor location, n (%)			0.912^#^
Upper pole	123 (36.3)	50 (34.2)	
Middle	106 (31.3)	47 (32.2)	
Lower pole	110 (32.4)	49 (33.6)	
Number of lesions, n (%)			0.766^#^
Single	197 (58.1)	82 (56.2)	
Multiple	142 (41.9)	64 (43.8)	
Distribution of lesions, n (%)			0.630^#^
Unilateral	230 (67.8)	103 (70.5)	
Bilateral	109 (32.2)	43 (29.5)	
Tumor size, cm, median (IQR)	1.60 (1.10, 2.30)	1.60 (1.20, 2.10)	0.987^*^
Irregular shape, n (%)			0.598^#^
Yes	79 (23.3)	38 (26.0)	
No	260 (76.7)	108 (74.0)	
Capsular invasion, n (%)			0.148^#^
Yes	146 (43.1)	74 (50.7)	
No	193 (56.9)	72 (49.3)	
Extraglandular invasion, n (%)			0.453^#^
Yes	105 (31.0)	51 (34.9)	
No	234 (69.0)	95 (65.1)	
Hypoechoic mass, n (%)			0.574^#^
Yes	285 (84.1)	119 (81.5)	
No	54 (15.9)	27 (18.5)	
Calcified foci, n (%)			0.696^#^
Yes	106 (31.3)	49 (33.6)	
No	233 (68.7)	97 (66.4)	
Doppler blood flow, n (%)			0.847^#^
Rich	71 (20.9)	34 (23.3)	
Little	191 (56.3)	80 (54.8)	
None	77 (22.7)	32 (21.9)	
Ki-67, n (%)			0.982^#^
<5%	264 (77.9)	113 (77.4)	
5-10%	71 (20.9)	31 (21.2)	
>10%	4 (1.2)	2 (1.4)	
P53, n (%)			0.890^#^
<5%	236 (69.6)	100 (68.5)	
≥5%	103 (30.4)	46 (31.5)	
CK-19, n (%)			0.862^#^
<25%	71 (20.9)	27 (18.5)	
25-50%	219 (64.6)	95 (65.1)	
50-75%	31 (9.1)	14 (9.6)	
>75%	18 (5.3)	10 (6.8)	
Harvested number of CLN, mean ± SD	8.23 ± 3.28	8.58 ± 3.21	0.280^$^
Metastatic number of CLN, mean ± SD	0	0	NA
Pathological subtype			0.935
C-PTC	312 (92.0)	133 (91.1)	
FV-PTC	14 (4.1)	7 (4.8)	
Other ^a^	13 (3.8)	6 (4.1)	

^#^For Chi-square; ^$^for independent sample t-test; ^*^For Mann–Whitney U test; IQR, inter-quartile range; SD, standard deviation; BMI, body mass index; CLN, central lymph nodes; NA, not applicable; C-PTC, classic PTC; FV-PTC, follicular variant PTC; PTC, papillary thyroid cancer; ^a^Other: including tall cell, columnar cell, and hobnail variant subtypes.

### Comparative clinicopathological characteristics of patients with and without SLNM in the training cohort

**Table 3** presents a comparison of clinicopathological characteristics between patients with and without SLNM in the training cohort, identifying associations between SLNM risk and factors such as age, tumor location, number of lesions, tumor size, capsular and extraglandular invasions, Ki-67, and P53 (all P < 0.05). Key clinicopathological parameters were standardized to a mean of zero and a standard deviation of one using Z-score normalization. These standardized metrics were then utilized to develop clinicopathological ML prediction models.

**Table 3:** Comparisons of the clinicopathological characteristics between the non-SLNM and SLNM groups.

Clinicopathological characteristics	Non-SLNM group (n=290)	SLNM group (n=49)	P value
Age, year, median (IQR)	43.00 (35.00, 49.00)	51.00 (41.00, 59.00)	<0.001^*^
Gender, n (%)			0.973^#^
Male	87 (30.0)	14 (28.6)	
Female	203 (70.0)	35 (71.4)	
BMI, kg/m^2^, median (IQR)	24.00 (20.90, 27.28)	22.70 (21.40, 26.80)	0.798^*^
Diabetes, n (%)			0.223^#^
Yes	32 (11.0)	9 (18.4)	
No	258 (89.0)	40 (81.6)	
Graves’ disease, n (%)			0.187_#_
Yes	2 (0.7)	2 (4.1)	
No	288 (99.3)	47 (95.9)	
Family history of thyroid cancer, n (%)			0.759^#^
Yes	25 (8.6)	3 (6.1)	
No	265 (91.4)	46 (93.9)	
History of Hashimoto’s thyroiditis, n (%)			0.650^#^
Yes	37 (12.8)	8 (16.3)	
No	253 (87.2)	41 (83.7)	
T stage of tumor, n (%)			0.445^#^
T1	14 (4.8)	3 (6.1)	
T2	166 (57.2)	26 (53.1)	
T3	88 (30.3)	13 (26.5)	
T4	22 (7.6)	7 (14.3)	
Tumor location, n (%)			<0.001^#^
Upper pole	93 (32.1)	30 (61.2)	
Middle	93 (32.1)	13 (26.5)	
Lower pole	104 (35.9)	6 (12.2)	
Number of lesions, n (%)			0.002^#^
Single	158 (54.5)	39 (79.6)	
Multiple	132 (45.5)	10 (20.4)	
Distribution of lesions, n (%)			0.282^#^
Unilateral	193 (66.6)	37 (75.5)	
Bilateral	97 (33.4)	12 (24.5)	
Tumor size, cm, median (IQR)	1.60 (1.10, 2.20)	2.60 (1.52, 3.00)	<0.001^*^
Irregular shape, n (%)			0.260^#^
Yes	64 (22.1)	15 (30.6)	
No	226 (77.9)	34 (69.4)	
Capsular invasion, n (%)			<0.001^#^
Yes	111 (38.3)	35 (71.4)	
No	179 (61.7)	14 (28.6)	
Extraglandular invasion, n (%)			0.002^#^
Yes	80 (27.6)	25 (51.0)	
No	210 (72.4)	24 (49.0)	
Hypoechoic mass, n (%)			0.897^#^
Yes	243 (83.8)	42 (85.7)	
No	47 (16.2)	7 (14.3)	
Calcified foci, n (%)			0.695^#^
Yes	89 (30.7)	17 (34.7)	
No	201 (69.3)	32 (65.3)	
Doppler blood flow, n (%)			0.342^#^
Rich	57 (19.7)	14 (28.6)	
Little	167 (57.6)	24 (49.0)	
None	66 (22.8)	11 (22.4)	
Ki-67, n (%)			0.004^#^
<5%	234 (80.7)	30 (61.2)	
5-10%	54 (18.6)	17 (34.7)	
>10%	2 (0.7)	2 (4.1)	
P53, n (%)			0.026^#^
<5%	209 (72.1)	27 (55.1)	
≥5%	81 (27.9)	22 (44.9)	
CK-19 (%)			0.184^#^
<25%	66 (22.8)	5 (10.2)	
25-50%	185 (63.8)	34 (69.4)	
50-75%	25 (8.6)	6 (12.2)	
>75%	14 (4.8)	4 (8.2)	
Harvested number of CLN, mean ± SD	8.19 ± 3.28	8.49 ± 3.29	0.554^$^
Metastatic number of CLN, mean ± SD	0	0	NA
Pathological subtype			0.995
C-PTC	267 (92.1)	45 (91.8)	
FV-PTC	12 (4.1)	2 (4.1)	
Other ^a^	11 (3.8)	2 (4.1)	

#For Chi-square; ^$^for independent sample t-test; ^*^For Mann–Whitney U test; SLNM, skip lymph node metastasis; IQR, inter-quartile range; SD, standard deviation; BMI, body mass index; CLN, central lymph nodes; NA, not applicable; C-PTC, classic PTC; FV-PTC, follicular variant PTC; PTC, papillary thyroid cancer; ^a^ Other: including tall cell, columnar cell, and hobnail variant subtypes.

### Radiomics analysis

**Figure 3:**
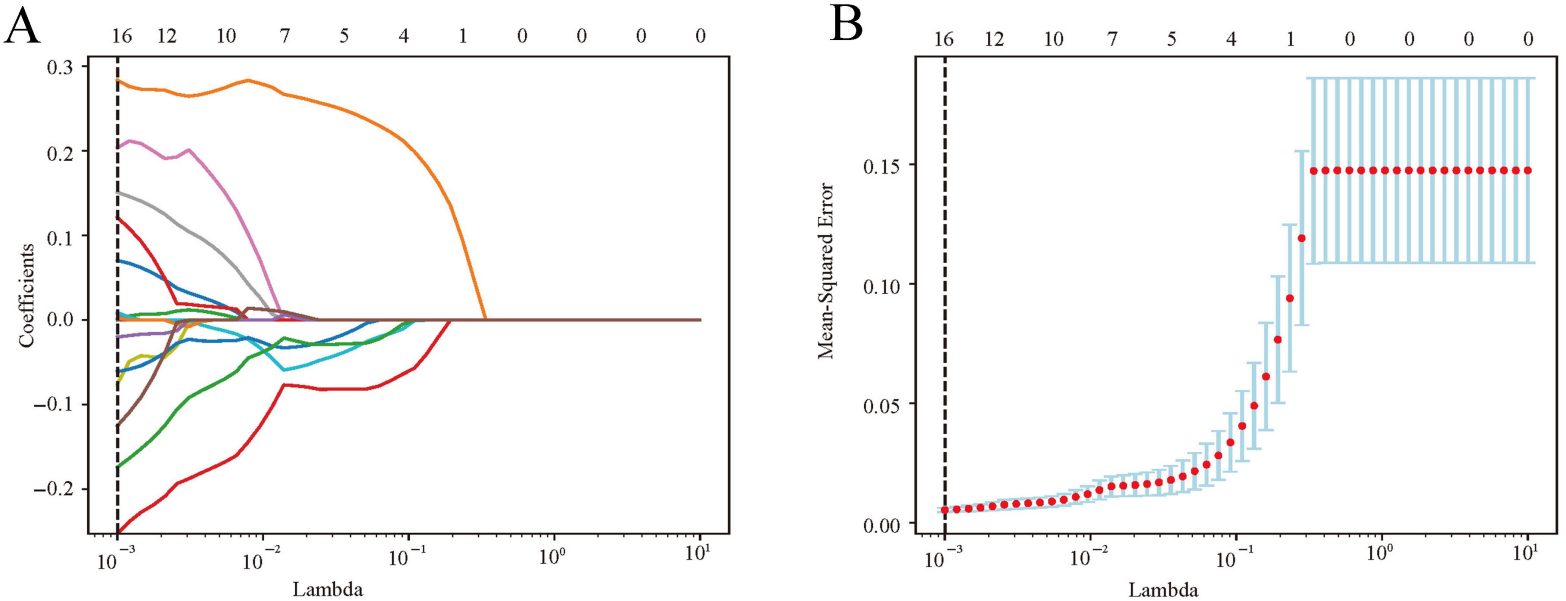
Radiomics feature selection via LASSO logistic regression. **(A)** LASSO coefficient profiles were plotted against the lambda values. **(B)** Repeat the 10-fold cross-validation process 50 times to identify the optimal penalization coefficient, lambda, in the LASSO model, yielding 16 nonzero coefficients. The red dots indicate the mean value of the target parameters. LASSO, least absolute shrinkage and selection operator.

In the training cohort, 1115 radiomics features were extracted and normalized from each elastography image, with ICCs ranging from 0.5 to 0.99. Notably, 981 features (88.0%) demonstrated an intra-observer ICC of ≥ 0.8 and were selected initially. Through a Student’s t-test, this set was refined to 22 potential predictors. Subsequently, a LASSO logistic regression model identified 16 optimal features correlated with SLNM, each distinguished by non-zero coefficients (**Figures 3A, B**).

### Model comparison for SLNM risk prediction

**Figure 4:**
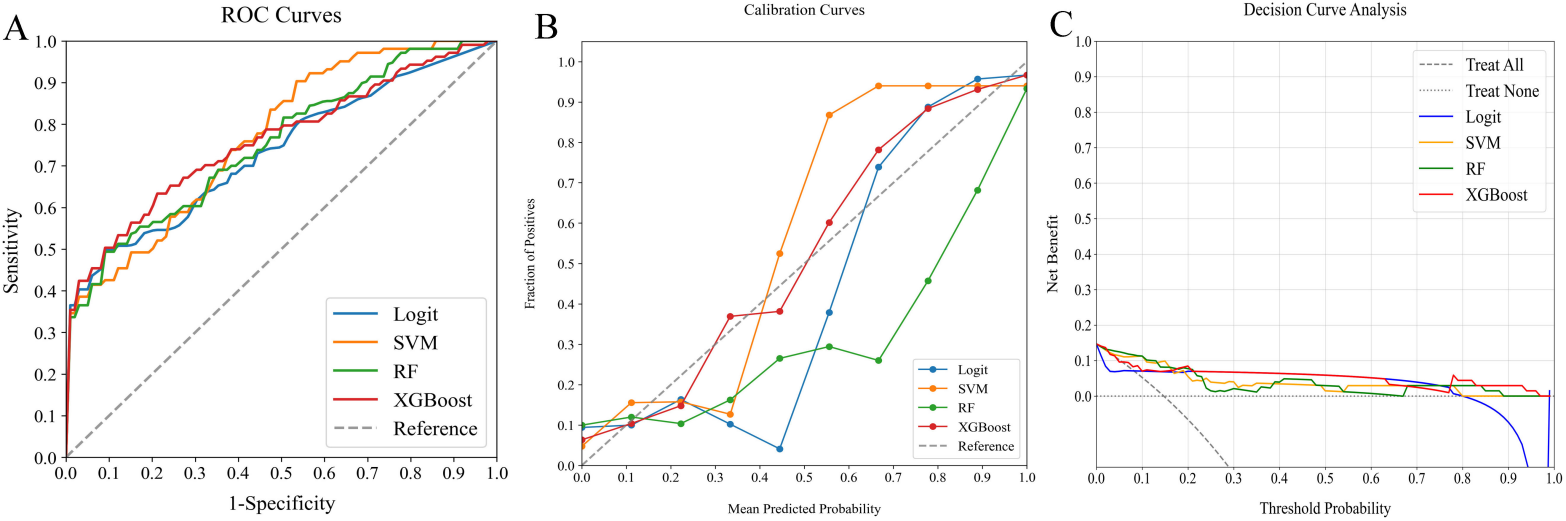
Comparative analysis of ML classifiers (Logit, SVM, RF, and XGBoost) using clinicopathological data: performance metrics including **(A)** ROC curves, **(B)** calibration plots, and **(C)** DCA. They achieved ROC-AUCs of 0.744, 0.769, 0.752, and 0.760, respectively. ML, machine learning; ROC, receiver operating characteristic; AUC, area under the curve; DCA, decision curve analysis; Logit, logistic regression; SVM, support vector machine; RF, random forest; XGBoost, extreme gradient boosting.

**Figure 5:**
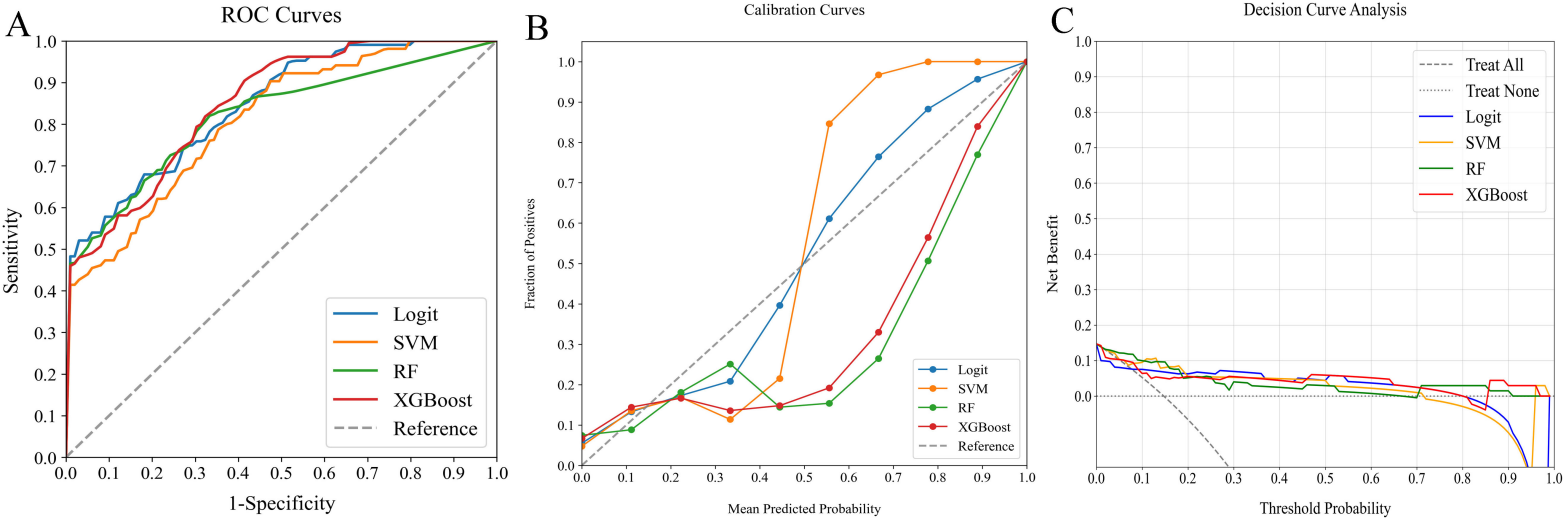
Comparative analysis of ML classifiers (Logit, SVM, RF, and XGBoost) using radiomics features: performance metrics including **(A)** ROC curves, **(B)** calibration plots, and **(C)** DCA. They achieved ROC-AUCs of 0.844, 0.809, 0.818, and 0.847, respectively. ML, machine learning; ROC, receiver operating characteristic; AUC, area under the curve; DCA, decision curve analysis; Logit, logistic regression; SVM, support vector machine; RF, random forest; XGBoost, extreme gradient boosting.

**Figure 6:**
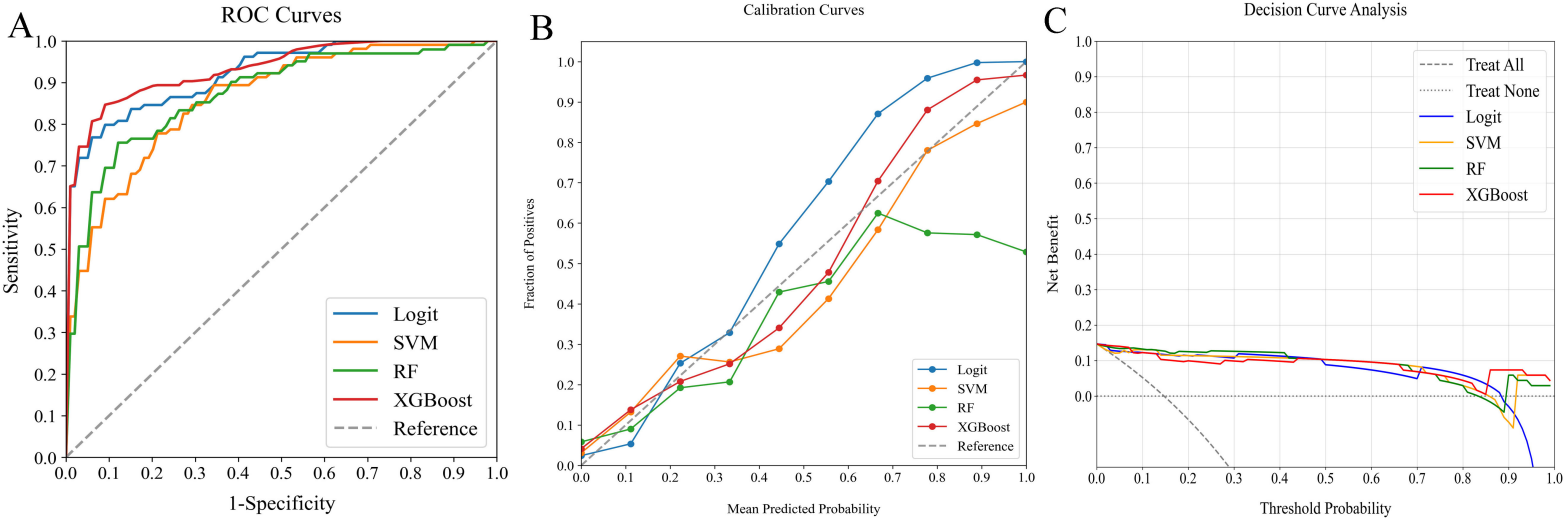
Comparative analysis of ML classifiers (Logit, SVM, RF, and XGBoost) on clinicopathological and radiomics data: performance metrics **(A)** ROC curves, **(B)** calibration plots, and **(C)** DCA. They achieved ROC-AUCs of 0.922, 0.861, 0.872, and 0.934, respectively. ML, machine learning; ROC, receiver operating characteristic; AUC, area under the curve; DCA, decision curve analysis; Logit, logistic regression; SVM, support vector machine; RF, random forest; XGBoost, extreme gradient boosting.

In our study, we assessed the efficacy of predictive models for evaluating SLNM risk in cN0 PTC patients using four ML classifiers: Logit, SVM, RF, and XGBoost. These models were applied to three datasets: clinicopathological, radiomics, and a combined dataset. **Table 4** systematically compares these models, and **Figures 4**–**6** depict their performance metrics, including ROC, calibration, and DCA curves. The results demonstrated that the clinicopathological-radiomics models, which integrate both clinicopathological and radiomics data, significantly outperformed the models based solely on clinicopathological data (AUC: 0.744–0.769) or radiomics data (AUC: 0.809–0.847), achieving AUCs between 0.861 and 0.934. This superiority was statistically confirmed by Delong’s test (all P < 0.05).

**Table 4:** Performance of ML classifiers for predicting SLNM risk in cN0 PTC patients using clinicopathological data, radiomics features, and combined datasets.

Data Type	ML classifier	AUC	Precision	Recall	F1 Score	Brier Score
Clinicopathological data	Logit	0.744	0.750	0.250	0.375	0.028
	SVM	0.769	0.651	0.451	0.622	0.026
	RF	0.752	0.667	0.333	0.444	0.047
	XGBoost	0.76	0.771	0.701	0.699	0.004
Radiomics feature	Logit	0.844	0.511	0.183	0.154	0.005
	SVM	0.809	0.512	0.233	0.412	0.034
	RF	0.818	0.655	0.333	0.500	0.052
	XGBoost	0.847	0.801	0.633	0.502	0.043
Combined clinicopathological and radiomics data	Logit	0.922	0.833	0.817	0.846	0.012
	SVM	0.861	0.800	0.813	0.801	0.007
	RF	0.872	0.812	0.803	0.799	0.040
	XGBoost	0.934	0.833	0.817	0.856	0.001

ML, machine learning; SLNM, skip lymph node metastasis; cN0, clinically node-negative; PTC, papillary thyroid cancer; AUC, area under the curve; Logit, logistic regression; SVM, support vector machine; RF, random forest; XGBoost, extreme gradient boosting.

In our evaluation of clinicopathological-radiomics models, XGBoost excelled, achieving the highest AUC score of 0.934 and showing superior calibration, particularly near the 60% threshold. Performance across all models was consistently demonstrated in DCA. XGBoost consistently outperformed in key metrics such as Precision, Recall, F1 Score, and Brier Score, highlighting its effectiveness. These findings establish XGBoost as the most suitable model for preoperative prediction of SLNM risk.

### Assessing ML model with the external verification cohort

**Figure 7:**
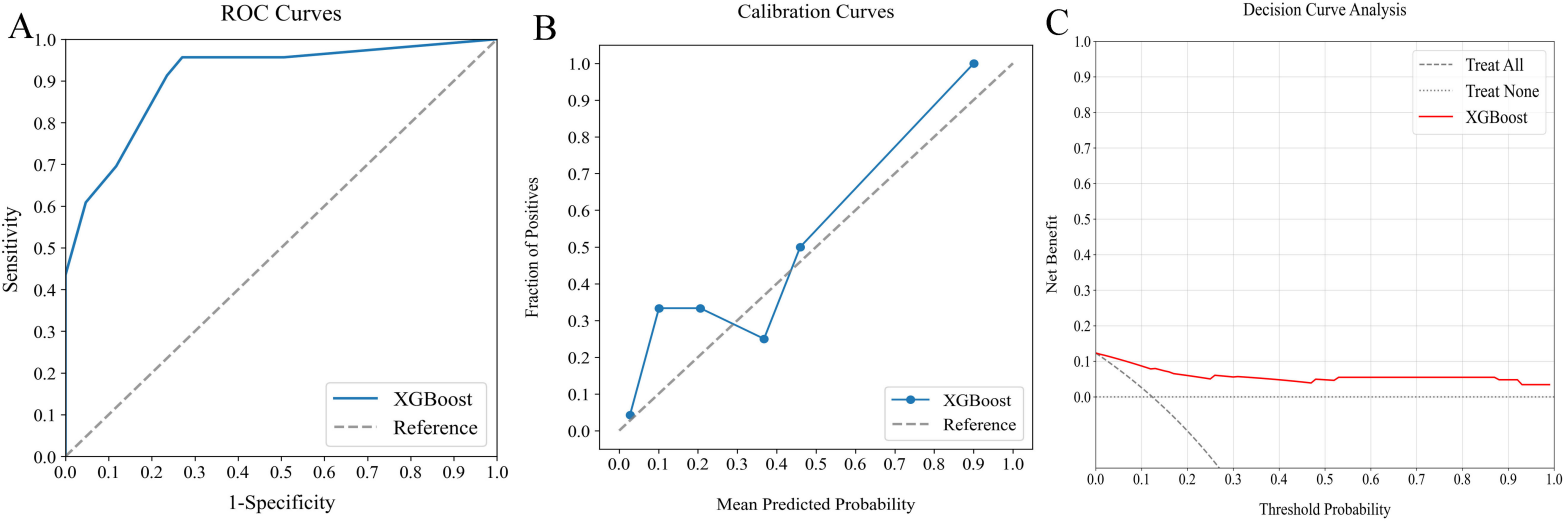
Evaluation of optimal ML model’s predictive performance with external verification cohort. **(A)** ROC curve (AUC = 0.907) indicating significant discriminative capacity, **(B)** calibration curve confirming strong agreement between predictions and observations, especially above 40%, and **(C)** DCA highlighting net clinical benefit across prediction probabilities. ML, machine learning; ROC, receiver operating characteristic; AUC, area under the curve; DCA, decision curve analysis.

The external verification cohort was employed to assess the predictive accuracy of the XGBoost model against actual SLNM outcomes using ROC, calibration, and DCA analyses (**Figure 7**). Although the model exhibited a slight performance dip compared to the training cohort, it maintained significant discriminative ability with an AUC of 0.907 (**Figure 7A**). The calibration curve showed strong alignment between predicted risks and observed frequencies, particularly for values above the 40% threshold (**Figure 7B**). Additionally, the DCA curve confirmed the model’s effectiveness by demonstrating substantial net benefits (**Figure 7C**). These results highlight the XGBoost model’s utility as an effective predictive tool for SLNM risk in clinical settings.

### Interpretation of the model

**Figure 8:**
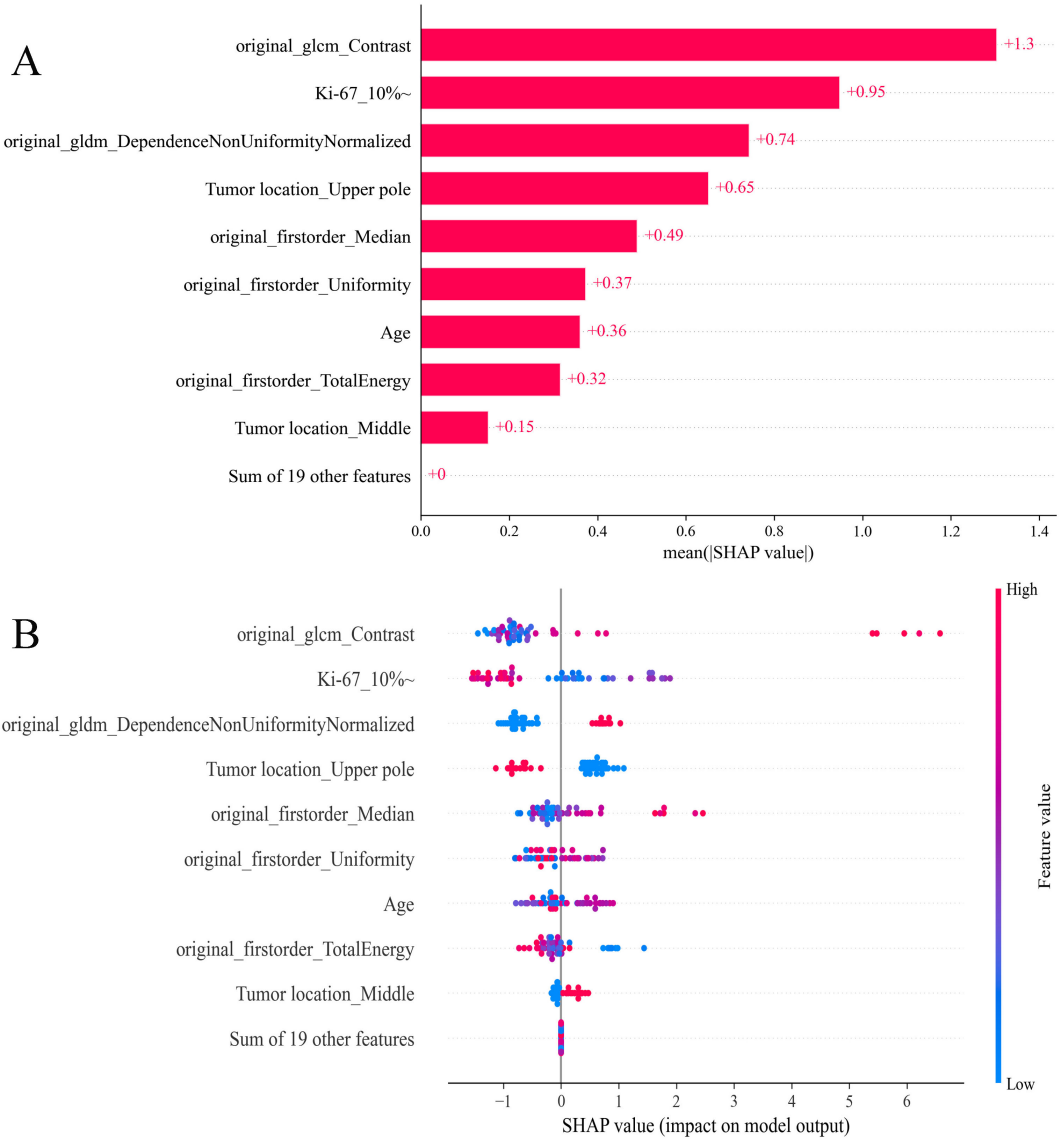
SHAP analysis of XGBoost model predicting SLNM risk in cN0 PTC patients: **(A)** feature significance ranking based on absolute mean SHAP values, **(B)** summary plot visualizing cumulative influence. SHAP, Shapley Additive Explanation; XGBoost, extreme gradient boosting; SLNM, skip lymph node metastasis; cN0, clinically node-negative; PTC, papillary thyroid cancer.

The SHAP analysis was used to decode the XGBoost model by quantifying the impact of each feature. This involved computing the absolute mean SHAP values, which helped prioritize features by importance. Notably, three radiomics features from elastography images, one clinical variable, and one pathological variable were identified as the most influential in the model (**Figure 8A**). A summary plot displayed the collective impact of these features through their SHAP values (**Figure 8B**). This visualization offered comprehensive insights into the contribution of each feature to individual patient predictions. Crucially, higher values of these top five features were associated with an increased risk of SLNM in cN0 PTC patients.

**Figure 9:**
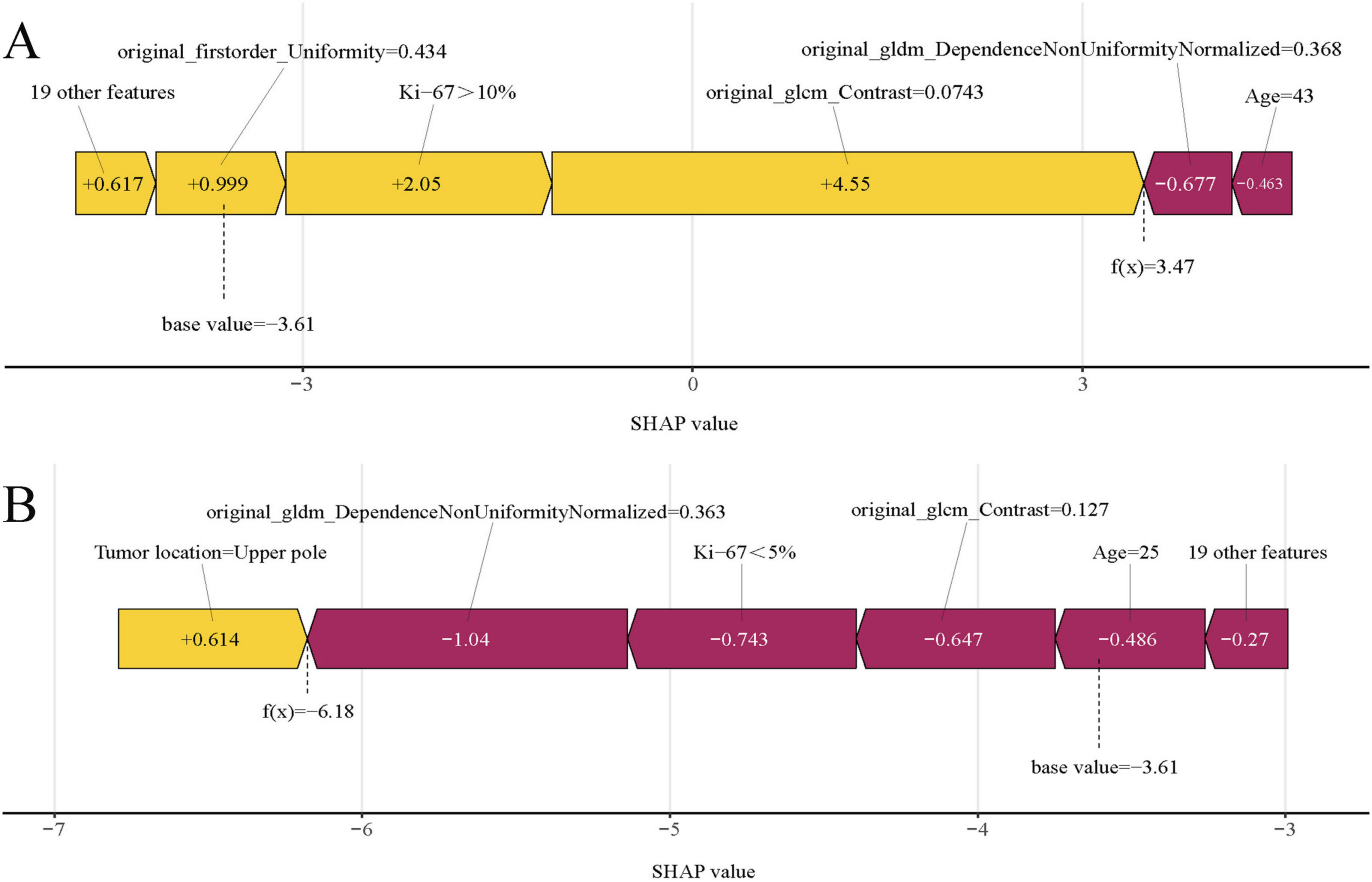
SHAP force plots illustrating individual prediction results: **(A)** for a patient with SLNM; **(B)** for a patient without SLNM. SHAP, Shapley Additive Explanations; SLNM, skip lymph node metastasis.

In predictive modeling, the SHAP force plot effectively illustrates how specific features influence individual patient outcomes (**Figure 9**). Yellow areas represent features that increase the likelihood of SLNM in cN0 PTC patients, while red areas represent features that decrease it. A wider color region indicates a more substantial impact. The value f(x) aggregates the SHAP values for each patient, with the base value reflecting the average SHAP value across all patients. The top panel shows an accurate SLNM prediction due to factors like Ki-67 >10% (**Figure 9A**). Conversely, the bottom panel accurately predicts a non-SLNM case, considering features such as Ki-67 <5% and age 25 years (**Figure 9B**). Utilizing XGBoost, this methodology effectively differentiates between patients at risk for SLNM or non-SLNM, facilitating personalized risk assessments.

## Discussion

In CLNM-negative cN0 patients, SLNM influences clinical staging and recurrence risk, underscoring the need for accurate preoperative prediction methods. This study aimed to meet this requirement by developing predictive models using four different ML classifiers based on clinicopathological data or elastography radiomics features. Our extensive evaluation, which included assessments of discriminative ability, calibration, and clinical utility, demonstrated that the XGBoost model combining both data types was most effective in predicting SLNM risk. Notably, integrating SHAP analysis enhanced the interpretability of the XGBoost model, pinpointing key clinicopathological and elastography radiomics features impacting SLNM risk. This research marks a significant advancement in the preoperative prediction of SLNM risk by merging clinicopathological data with elastography radiomics through ML models, paving the way for more accurate individual risk assessments.

In 2020, the American Association of Endocrine Surgeons updated the “Guidelines for the Definitive Surgical Management of Thyroid Disease in Adults,” advising against preventive neck lymph-node dissection for patients in T1, T2, and cN0 stages (30). However, the appropriateness of this conservative approach for Chinese patients remains debated. Yang P et al. argue there is insufficient evidence to standardize this method in China (31). Research shows that prophylactic CLND can safely prevent long-term metastasis and recurrence of thyroid cancer in PTC patients, even when lymph nodes show no signs of infiltration or metastasis (32, 33). In our study, we conducted CLND on PTC patients. Despite this, detecting SLNM with CLND remains challenging and may escape early ultrasonography due to atypical imaging features, potentially leading to misclassification of individuals as low-risk (15). Patients harboring occult SLNM often face increased risks of postoperative disease progression and potentially adverse outcomes, such as the need for additional surgeries (34). Accurate preoperative identification of these patients could significantly reduce the incidence of postoperative metastasis in cN0 PTC, thereby improving prognosis (34, 35). Most studies on SLNM have focused on cN+ patients, often excluding cN0 PTC patients (13, 15, 36). Although Yang et al. enrolled cN0 PTC patients in their study, they categorized all as non-SLNM without conducting postoperative follow-ups, thus overlooking the presence of occult SLNM in this group (37). Recently, Li et al. (38) enrolled cN0 PTC patients and conducted postoperative follow-ups. Based on these outcomes and pathology reports, cN0 patients with postoperative LLNM were categorized as having SLNM. However, their analysis was limited to clinical characteristics, omitting pathological variables and elastography radiomics. Furthermore, they relied on traditional linear predictive models rather than more effective ML techniques, compromising the precision of their predictions.

Several factors may contribute to postoperative LLNM in cN0 PTC patients: 1) Occult SLNM might exist prior to surgery, leading to LLNM through residual tumor cells in the LLNs (39); or 2) Following CLND, if the lymphatic pathways to CLNs are obstructed, tumor cells from residual thyroid tissue may metastasize to LLNs along the lymphatics near the upper pole of the thyroid vessels (40). Consequently, we posit that postoperative LLNM in cN0 PTC patients, particularly those who underwent CLND, is primarily attributed to SLNM. This study aims to detect occult SLNM through postoperative follow-up, explore risk factors for SLNM, and develop a predictive model to assess the likelihood of SLNM. In our study, the distributions of SLNM are focused on Levels II and III, which may relate to the cancerous nodule being located at the upper pole of the gland. Therefore, when a PTC nodule is located at the upper pole of the thyroid, it is essential to consider the presence of metastases in Level III of the lateral neck, while also remaining highly vigilant about the possibility of direct metastasis to Level II.

In our study, we selected ML models for their proficiency in handling complex, non-linear relationships between variables, surpassing traditional linear predictive methods (41). We evaluated four ML models using both clinicopathological and radiomics data. All models demonstrated adequate calibration and clinical utility, but their discriminative abilities varied significantly. Notably, models integrating clinicopathological with radiomics data exhibited the most effective prediction of SLNM, showing enhanced discrimination capabilities. This advantage likely stems from the comprehensive utilization of both clinicopathological and radiomics features, providing a broader analytical base compared to models that rely solely on one data type. This comprehensive feature integration likely explains the observed differences in predictive performance.

In our selection of ML models, XGBoost stood out as the most effective clinicopathological-radiomics model, maintaining high accuracy throughout external validation. To address the interpretability challenges associated with complex ML models, we employed the SHAP methodology. This approach clarifies the decision-making process on a cohort basis, complemented by intuitive visualizations. This allows for a detailed understanding of how individual variables impact predictions, thereby fostering trust in AI among clinicians (42, 43). Our study identified five principal predictors of SLNM risk: three radiomics features derived from elastography imaging, one clinical variable, and one pathological variable. The significance of elastography radiomics features was anticipated, reflecting their established correlation with SLNM. The radiomics features offer a more comprehensive and objective assessment than traditional imaging methods alone. Although the biological significance of certain texture features might seem abstract initially, they are crucial for understanding the complex attributes of thyroid nodules that transcend basic parameters such as shape and size. Additionally, the location of the tumor was confirmed as crucial clinical predictors. Notably, in our study, tumors located in the upper pole of the gland were present in 61.2% of cases in the SLNM group, a rate significantly higher than the 32.1% observed in the non-SLNM group. This finding aligns with the research conducted by Wang et al. (36) (SLNM group vs. non-SLNM group: 63.6% vs. 19.2%) and Weng et al. (44) (SLNM group vs. non-SLNM group: 53.0% vs. 24.4%). A likely explanation is that lymph from the upper pole of the thyroid primarily drains into the venous system via lateral cervical lymph nodes, following the lymphatic vessels that run alongside the superior thyroid artery (40, 45). Consequently, tumor cells in the upper pole are more prone to spreading to the LLNs through the ascending lymphatic vessels, increasing the risk of SLNM. Additionally, FNAB is a safe, cost-effective, and straightforward procedure that can help avoid invasive and potentially unnecessary surgeries for patients with thyroid swellings. Achieving an accurate pre-operative diagnosis of thyroid lesions remains a significant challenge for clinicians, making FNAB crucial as a diagnostic tool for thyroid carcinoma. In our study, we confirmed that a Ki-67 index greater than 10% in FNAB samples is associated with an increased risk of SLNM. Additionally, combining SHAP with XGBoost provides detailed insights into how variables affect outcomes, proving invaluable for predicting SLNM. This integration enhances the role of machine learning in clinical decision-making and improves patient outcomes. FNAB is a safe, cost-effective, and straightforward procedure that plays a crucial role in achieving accurate preoperative diagnoses of thyroid lesions and serves as an essential diagnostic tool for thyroid carcinoma (46). Additionally, a meta-analysis indicates that Ki-67 could influence the prognosis of thyroid cancer patients (47). However, the association between Ki-67 levels in preoperative settings and SLNM in PTC remains unclear. Our study confirmed that a preoperative Ki-67 >10% in FNA samples increases the risk of SLNM. Utilizing SHAP analysis, the XGBoost model provides detailed insights into how variables influence outcomes, proving invaluable for predicting SLNM. This enhances the role of ML in clinical decision-making and contributes to improved patient outcomes.

Our study suggests that this ML model can revolutionize management practices in several key areas. Firstly, it recommends either prophylactic lateral neck dissections or adjusting imaging follow-up intervals for patients at high risk of SLNM, enabling more tailored treatment strategies. Secondly, the model assists novice clinicians by directing patients likely to have SLNM to more seasoned specialists, thereby mitigating risks tied to clinical inexperience. Lastly, other clinicians can enter clinicopathological and elastography radiomics data into our XGBoost ML models for sharp clinical forecasts. Additionally, the model offers a SHAP force plot that delineates the influence of each variable on the outcomes, thus boosting both diagnostic precision and insight.

Our study identified three main limitations. Firstly, due to its retrospective nature and limited sample size, we could not evaluate lymphatic angioinvasion or conduct external validation to enhance our model’s performance. Secondly, a brief follow-up period may have overlooked postoperative metastases in patients with occult SLNM. Lastly, if applied across more institutions, variations in elastography settings could impact the extraction of radiomic features, potentially affecting the effectiveness of our ML models. Despite these challenges, our research confirmed the potential of clinicopathological-radiomics ML models for predicting SLNM in cN0 PTC patients. Future studies should focus on multi-center, prospective designs with larger cohorts and include inter-rater reliability tests to improve the model’s reliability and generalizability.

Micrometastatic lymph nodes in the CLN occur in 20% to 50% of patients with PTC, and rates can reach as high as 90% (48, 49). Preoperative ultrasound, however, only detects CLN with 30–55% accuracy and often misses lymph nodes under 5 mm in diameter (5–7). Even with CLND, sampling may be inadequate, leading to potential underdiagnosis (cN0) and underestimation of metastatic risk, despite the presence of SLNM. This underdiagnosis can significantly influence treatment decisions. Although prophylactic lateral neck dissection is not standard for cN0 PTC (2), undetected LNM may require additional interventions (8–12). Our evaluation highlights the XGBoost model, which integrates elastography radiomics and clinicopathological data, as the most effective ML approach for the prediction of SLNM in cN0 PTC patients with an increased risk of LNM. This innovative model significantly enhances the accuracy of risk assessments for SLNM, enabling personalized treatments that could reduce postoperative metastases in these patients.

## Data Availability

The original contributions presented in the study are included in the article/**Supplementary Material**. Further inquiries can be directed to the corresponding authors.

## References

[1] Liu X Fu Q Bian X Fu Y Xin J Liang N. Long Non-Coding RNA MAPK8IP1P2 Inhibits Lymphatic Metastasis of Thyroid Cancer by Activating Hippo Signaling via Sponging miR-146b-3p. Front Oncol. (2020) 10:600927. doi: 10.3389/fonc.2020.600927 33489905 PMC7817949

[2] Haugen BR Alexander EK Bible KC Doherty GM Mandel SJ Nikiforov YE. 2015 American thyroid association management guidelines for adult patients with thyroid nodules and differentiated thyroid cancer: the American thyroid association guidelines task force on thyroid nodules and differentiated thyroid cancer. Thyroid. (2016) 26:1–133. doi: 10.1089/thy.2015.0020 26462967 PMC4739132

[3] Shi L Song H Zhu H Li D Zhang N. Pattern, predictors, and recurrence of cervical lymph node metastases in papillary thyroid cancer. Contemp Oncol (Pozn). (2013) 17:504–9. doi: 10.5114/wo.2013.38910 PMC3934041 24592137

[4] Lei J Zhong J Jiang K Li Z Gong R Zhu J. Skip lateral lymph node metastasis leaping over the central neck compartment in papillary thyroid carcinoma. Oncotarget. (2017) 8:27022–33. doi: 10.18632/oncotarget.15388 PMC5432315 28223546

[5] Stulak JM Grant CS Farley DR Thompson GB van Heerden JA Hay ID. Value of preoperative ultrasonography in the surgical management of initial and reoperative papillary thyroid cancer. Arch Surg. (2006) 141:489–94. doi: 10.1001/archsurg.141.5.489 16702521

[6] Hartl DM Leboulleux S Al Ghuzlan A Baudin E Chami L Schlumberger M. Optimization of staging of the neck with prophylactic central and lateral neck dissection for papillary thyroid carcinoma. Ann Surg. (2012) 255:777–83. doi: 10.1097/SLA.0b013e31824b7b68 22418010

[7] Shin LK Olcott EW Jeffrey RB Desser TS. Sonographic evaluation of cervical lymph nodes in papillary thyroid cancer. Ultrasound Q. (2013) 29:25–32. doi: 10.1097/RUQ.0b013e31827c7a9e 23358214

[8] Mitchell AL Gandhi A Scott-Coombes D Perros P. Management of thyroid cancer: United Kingdom National Multidisciplinary Guidelines. J Laryngol Otol. (2016) 130:S150–s160. doi: 10.1017/s0022215116000578 27841128 PMC4873931

[9] Hu D Zhou J He W Peng J Cao Y Ren H. Risk factors of lateral lymph node metastasis in cN0 papillary thyroid carcinoma. World J Surg Oncol. (2018) 16:30. doi: 10.1186/s12957-018-1336-3 29439716 PMC5811970

[10] Roh JL Park JY Rha KS Park CI. Is central neck dissection necessary for the treatment of lateral cervical nodal recurrence of papillary thyroid carcinoma? Head Neck. (2007) 29:901–6. doi: 10.1002/hed.20606 17405173

[11] Kawabe J Higashiyama S Sougawa M Yoshida A Kotani K. Usefulness of stereotactic radiotherapy using cyberKnife for recurrent lymph node metastasis of differentiated thyroid cancer. Case Rep Endocrinol. (2017) 2017:7956726. doi: 10.1155/2017/7956726 28396808 PMC5370468

[12] Liu FH Kuo SF Hsueh C Chao TC Lin JD. Postoperative recurrence of papillary thyroid carcinoma with lymph node metastasis. J Surg Oncol. (2015) 112:149–54. doi: 10.1002/jso.23967 PMC5034820 26175314

[13] Attard A Paladino NC Lo Monte AI Falco N Melfa G Rotolo G. Skip metastases to lateral cervical lymph nodes in differentiated thyroid cancer: a systematic review. BMC Surg. (2019) 18:112. doi: 10.1186/s12893-018-0435-y 31074393 PMC7402576

[14] Jin WX Jin YX Ye DR Zheng ZC Sun YH Zhou XF. Predictive factors of skip metastasis in papillary thyroid cancer. Med Sci Monit. (2018) 24:2744–9. doi: 10.12659/msm.907357 PMC5952804 29722351

[15] Nie X Tan Z Ge M. Skip metastasis in papillary thyroid carcinoma is difficult to predict in clinical practice. BMC Cancer. (2017) 17:702. doi: 10.1186/s12885-017-3698-2 29070029 PMC5657116

[16] Yao X Meng Y. Value of ultrasound combined with immunohistochemistry evaluation of central lymph node metastasis for the prognosis of papillary thyroid carcinoma. Cancer Manag Res. (2020) 12:8787–99. doi: 10.2147/cmar.s265756 PMC7519832 33061575

[17] Moon JH Kim YI Lim JA Choi HS Cho SW Kim KW. Thyroglobulin in washout fluid from lymph node fine-needle aspiration biopsy in papillary thyroid cancer: large-scale validation of the cutoff value to determine Malignancy and evaluation of discrepant results. J Clin Endocrinol Metab. (2013) 98:1061–8. doi: 10.1210/jc.2012-3291 23393171

[18] Grani G Fumarola A. Thyroglobulin in lymph node fine-needle aspiration washout: a systematic review and meta-analysis of diagnostic accuracy. J Clin Endocrinol Metab. (2014) 99:1970–82. doi: 10.1210/jc.2014-1098 24617715

[19] Liu RB Zhou DL Xu BH Yang XH Liu Q Zhang X. Comparison of the diagnostic performances of US-guided fine needle aspiration cytology and thyroglobulin measurement for lymph node metastases in patients with differentiated thyroid carcinoma: a meta-analysis. Eur Radiol. (2021) 31:2903–14. doi: 10.1007/s00330-020-07400-9 33125564

[20] Zhao CK Xu HX Xu JM Sun CY Chen W Liu BJ. Risk stratification of thyroid nodules with Bethesda category III results on fine-needle aspiration cytology: The additional value of acoustic radiation force impulse elastography. Oncotarget. (2017) 8:1580–92. doi: 10.18632/oncotarget.13685 PMC5352079 27906671

[21] Xu JM Chen YJ Dang YY Chen M. Association between preoperative US, elastography features and prognostic factors of papillary thyroid cancer with BRAF(V600E) mutation. Front Endocrinol (Lausanne). (2019) 10:902. doi: 10.3389/fendo.2019.00902 32038479 PMC6987316

[22] Feng ST Jia Y Liao B Huang B Zhou Q Li X. Preoperative prediction of microvascular invasion in hepatocellular cancer: a radiomics model using Gd-EOB-DTPA-enhanced MRI. Eur Radiol. (2019) 29:4648–59. doi: 10.1007/s00330-018-5935-8 30689032

[23] Kagiyama N Shrestha S Cho JS Khalil M Singh Y Challa A. A low-cost texture-based pipeline for predicting myocardial tissue remodeling and fibrosis using cardiac ultrasound. EBioMedicine. (2020) 54:102726. doi: 10.1016/j.ebiom.2020.102726 32268274 PMC7139137

[24] Li W Huang Y Zhuang BW Liu GJ Hu HT Li X. Multiparametric ultrasomics of significant liver fibrosis: A machine learning-based analysis. Eur Radiol. (2019) 29:1496–506. doi: 10.1007/s00330-018-5680-z PMC6510867 30178143

[25] Ge XY Lan ZK Lan QQ Lin HS Wang GD Chen J. Diagnostic accuracy of ultrasound-based multimodal radiomics modeling for fibrosis detection in chronic kidney disease. Eur Radiol. (2023) 33:2386–98. doi: 10.1007/s00330-022-09268-3 PMC10017610 36454259

[26] Jiang T Gradus JL Rosellini AJ. Supervised machine learning: A brief primer. Behav Ther. (2020) 51:675–87. doi: 10.1016/j.beth.2020.05.002 PMC7431677 32800297

[27] Meng F Wu Q Zhang W Hou S. Application of interpretable machine learning models based on ultrasonic radiomics for predicting the risk of fibrosis progression in diabetic patients with nonalcoholic fatty liver disease. Diabetes Metab Syndr Obes. (2023) 16:3901–13. doi: 10.2147/dmso.s439127 PMC10700041 38077485

[28] Wang W Dai J Li J Du X. Predicting postoperative rehemorrhage in hypertensive intracerebral hemorrhage using noncontrast CT radiomics and clinical data with an interpretable machine learning approach. Sci Rep. (2024) 14:9717. doi: 10.1038/s41598-024-60463-2 38678066 PMC11055901

[29] Zuo D Yang L Jin Y Qi H Liu Y Ren L. Machine learning-based models for the prediction of breast cancer recurrence risk. BMC Med Inform Decis Mak. (2023) 23:276. doi: 10.1186/s12911-023-02377-z 38031071 PMC10688055

[30] Patel KN Yip L Lubitz CC Grubbs EG Miller BS Shen W. The american association of endocrine surgeons guidelines for the definitive surgical management of thyroid disease in adults. Ann Surg. (2020) 271:e21–93. doi: 10.1097/sla.0000000000003580 32079830

[31] Yang P Li J. Effect of prophylactic central lymph node dissection on locoregional recurrence in patients with papillary thyroid microcarcinoma. Int J Endocrinol. (2021) 2021:8270622. doi: 10.1155/2021/8270622 34819955 PMC8608519

[32] Zhao H Li H. Meta-analysis of ultrasound for cervical lymph nodes in papillary thyroid cancer: Diagnosis of central and lateral compartment nodal metastases. Eur J Radiol. (2019) 112:14–21. doi: 10.1016/j.ejrad.2019.01.006 30777203

[33] Yan B Hou Y Chen D He J Jiang Y. Risk factors for contralateral central lymph node metastasis in unilateral cN0 papillary thyroid carcinoma: A meta-analysis. Int J Surg. (2018) 59:90–8. doi: 10.1016/j.ijsu.2018.09.004 30342280

[34] Xu JJ Yu E McMullen C Pasternak J Brierley J Tsang R. Patterns of regional recurrence in papillary thyroid cancer patients with lateral neck metastases undergoing neck dissection. J Otolaryngol Head Neck Surg. (2017) 46:43. doi: 10.1186/s40463-017-0221-3 28569186 PMC5452602

[35] McNamara WF Wang LY Palmer FL Nixon IJ Shah JP Patel SG. Pattern of neck recurrence after lateral neck dissection for cervical metastases in papillary thyroid cancer. Surgery. (2016) 159:1565–71. doi: 10.1016/j.surg.2016.02.005 PMC5138026 26994486

[36] Wang W Yang Z Ouyang Q. A nomogram to predict skip metastasis in papillary thyroid cancer. World J Surg Oncol. (2020) 18:167. doi: 10.1186/s12957-020-01948-y 32669128 PMC7366301

[37] Yang Z Heng Y Zhao Q Cao Z Tao L Qiu W. A specific predicting model for screening skip metastasis from patients with negative central lymph nodes metastasis in papillary thyroid cancer. Front Endocrinol (Lausanne). (2021) 12:743900. doi: 10.3389/fendo.2021.743900 34659126 PMC8515125

[38] Li F Zhou FJ Zhu TW Qiu HL Zhang XT Ruan BW. Nomogram for predicting skip metastasis in cN0 papillary thyroid cancer patients at increased risk of lymph node metastasis. Adv Clin Exp Med. (2023) 32:753–61. doi: 10.17219/acem/157240 36603142

[39] Kliseska E Makovac I. Skip metastases in papillary thyroid cancer. Coll Antropol. (2012) 36 Suppl 2:59–62. 23397756

[40] Likhterov I Reis LL Urken ML. Central compartment management in patients with papillary thyroid cancer presenting with metastatic disease to the lateral neck: Anatomic pathways of lymphatic spread. Head Neck. (2017) 39:853–9. doi: 10.1002/hed.24568 28252836

[41] Uddin S Khan A Hossain ME Moni MA. Comparing different supervised machine learning algorithms for disease prediction. BMC Med Inform Decis Mak. (2019) 19:281. doi: 10.1186/s12911-019-1004-8 31864346 PMC6925840

[42] Nohara Y Matsumoto K Soejima H Nakashima N. Explanation of machine learning models using shapley additive explanation and application for real data in hospital. Comput Methods Programs Biomed. (2022) 214:106584. doi: 10.1016/j.cmpb.2021.106584 34942412

[43] Xue B Li D Lu C King CR Wildes T Avidan MS. Use of machine learning to develop and evaluate models using preoperative and intraoperative data to identify risks of postoperative complications. JAMA Netw Open. (2021) 4:e212240. doi: 10.1001/jamanetworkopen.2021.2240 33783520 PMC8010590

[44] Weng HY Yan T Qiu WW Fan YB Yang ZL. The prognosis of skip metastasis in papillary thyroid microcarcinoma is better than that of continuous metastasis. J Clin Endocrinol Metab. (2022) 107:1589–98. doi: 10.1210/clinem/dgac107 35213704

[45] Lee YS Shin SC Lim YS Lee JC Wang SG Son SM. Tumor location-dependent skip lateral cervical lymph node metastasis in papillary thyroid cancer. Head Neck. (2014) 36:887–91. doi: 10.1002/hed.23391 23733708

[46] Abou-Foul AK Muzaffar J Diakos E Best JE Momtahan N Jayaram S. Correlation between thyroid fine needle aspiration cytology and postoperative histology: A 10-year single-centre experience. Cureus. (2021) 13:e14504. doi: 10.7759/cureus.14504 34007757 PMC8123937

[47] Pan DH Wen DY Luo YH Chen G Yang H Chen JQ. The diagnostic and prognostic values of Ki-67/MIB-1 expression in thyroid cancer: a meta-analysis with 6,051 cases. Onco Targets Ther. (2017) 10:3261–76. doi: 10.2147/ott.s135593 PMC5505609 28740401

[48] Noguchi S Noguchi A Murakami N. Papillary carcinoma of the thyroid. I. Developing pattern of metastasis. Cancer. (1970) 26:1053–60. doi: 10.1002/1097-0142(197011)26:5<1053::aid-cncr2820260513>3.0.co;2-x 5476786

[49] Cooper DS Doherty GM Haugen BR Kloos RT Lee SL Mandel SJ. Revised American Thyroid Association management guidelines for patients with thyroid nodules and differentiated thyroid cancer. Thyroid. (2009) 19:1167–214. doi: 10.1089/thy.2009.0110 19860577

