# Supplementary material for: Interpretable machine learning models for predicting skip metastasis in cN0 papillary thyroid cancer based on clinicopathological and elastography radiomics features
Source: Front Oncol. 2025 Jan 7;14:1457660. doi: 10.3389/fonc.2024.1457660 (PMC11758178; doi:10.3389/fonc.2024.1457660)
Supplement: Supplementary file 1 [file DataSheet1.docx]

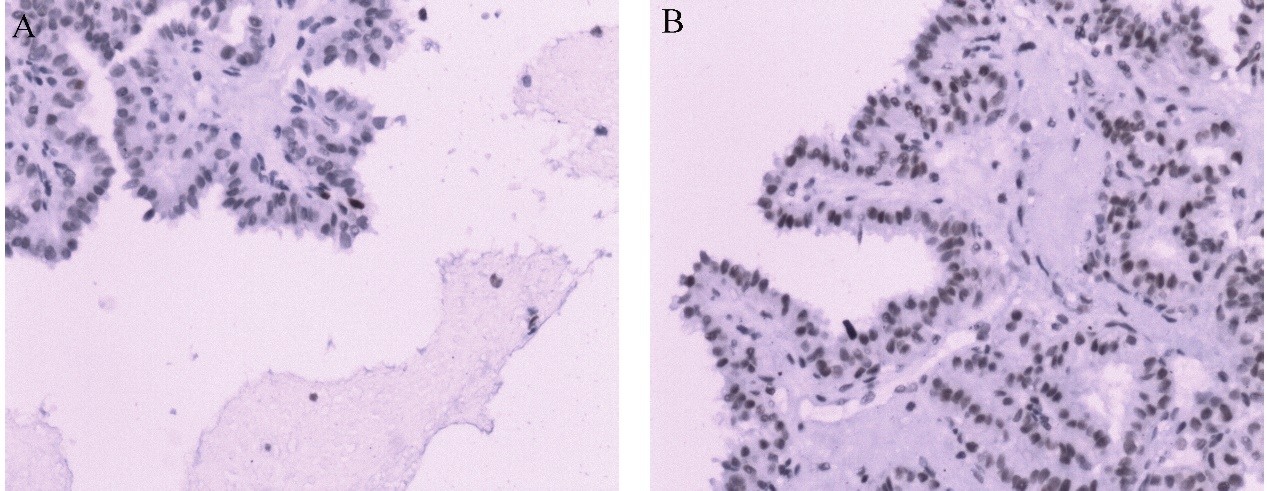


**Supplementary Figure 1**. Representative report of Ki-67 (A) and P53 (B) expression levels at 400x magnification.
