# Supplementary material for: Interpretable machine learning models for predicting skip metastasis in cN0 papillary thyroid cancer based on clinicopathological and elastography radiomics features
Source: Front Oncol. 2025 Jan 7;14:1457660. doi: 10.3389/fonc.2024.1457660 (PMC11758178; doi:10.3389/fonc.2024.1457660)
Supplement: Supplementary file 2 [file DataSheet2.docx]

**Code for Selection of Clinicopathological Features**

install.packages (readr)

library(readr)

data<-read_csv()

x1<-c()

x2<-c()

install.packages("tableone")

library(tableone)

table1<-CreateTableOne(vars=c(x1,x2),

data=data,

factorVars = x2,

strata='Group',addOverall = F)

results1<-print(table1,showAllLevels = F)

write.csv(results1,"result1.csv")

table2<-CreateTableOne(vars=c(x1,x2),

data=data,

factorVars = x2,

strata='Group',addOverall = F)

results2<-print(table2,showAllLevels = T,

nonnormal=x1)

write.csv(results2,"result2.csv")

**Code for Selection of Radiomics Features**

import pandas as pd

import numpy as np

from sklearn.utils import shuffle

from sklearn.preprocessing import StandardScaler

from sklearn.linear_model import LassoCV

from sklearn.model_selection import train_test_split,cross_val_score,KFold,RepeatedKFold,GridSearchCV

from scipy.stats import pearsonr, ttest_ind, levene

from sklearn.ensemble import RandomForestClassifier

from sklearn import svm

xlsx1_filePath='C:/Users/Lenovo/Desktop/data1.xlsx'

xlsx2_filePath='C:/Users/Lenovo/Desktop/data2.xlsx'

data_1=pd.read_excel(xlsx1_filePath)

data_2=pd.read_excel(xlsx2_filePath)

rows_1,_=data_1.shape

rows_2,_=data_2.shape

data_1.insert(0,'label',[0]*rows_1)

data_2.insert(0,'label',[1]*rows_2)

data=pd.concat([data_1,data_2])

data=shuffle(data)

data=data.fillna(0)

x=data[data.columns[1:]]

y=data['label']

#Check and handle non-convertible values

for col in x.columns:

try:

x[col].astype(np.float64)

except ValueError:

#Here, you can choose how to handle the non-convertible values.

#For example, replace them with 0 or NaN:

x[col]=pd.to_numeric(x[col],errors='coerce').fillna(0)

colNames=x.columns

x=x.astype(np.float64)

x=StandardScaler().fit_transform(x)

x=pd.DataFrame(x)

x.columns=colNames

from sklearn.preprocessing import MinMaxScaler

res=MinMaxScaler().fit_transform(data)

print(res)

counts=0

index=[]

data_1=data_1.apply(pd.to_numeric, errors='coerce').dropna()

data_2=data_2.apply(pd.to_numeric, errors='coerce').dropna()

for colName in data.columns[1:]:

#Ensure the column exists in both dataframes

if colName in data_1.columns and colName in data_2.columns:

#Convert columns to numeric and drop NaN values

data_1_col=pd.to_numeric(data_1[colName],errors='coerce').dropna()

data_2_col=pd.to_numeric(data_2[colName],errors='coerce').dropna()

if levene(data_1_col,data_2_col)[1]>0.05:

if ttest_ind(data_1_col,data_2_col)[1]<0.05:

#counts+=1

index.append(colName)

else:

if ttest_ind(data_1_col,data_2_col,equal_var=False)[1]<0.05:

#counts+=1

index.append(colName)

print(len(index))

if 'label'not in index:index=['label']+index

data_1=data_1[index]

data_2=data_2[index]

data=pd.concat([data_1,data_2])

data=shuffle(data)

data.index=range(len(data))

x=data[data.columns[1:]]

y=data['label']

x=x.apply(pd.to_numeric,errors='ignore')

colNames=x.columns

x=x.fillna(0)

x=x.astype(np.float64)

x=StandardScaler().fit_transform(x)

x=pd.DataFrame(x)

x.columns=colNames

alphas=np.logspace(-3,1,50)

model_lassoCV=LassoCV(alphas=alphas,cv=10,max_iter=100000).fit(x,y)

print(model_lassoCV.alpha_)

coef=pd.Series(model_lassoCV.coef_,index=x.columns)

print("Lasso picked"+str(sum(coef!=0))+"variables and eliminated the other"+str(sum(coef==0)))

index=coef[coef!=0].index

x=x[index]

#x.head()

print(coef[coef!=0])

x_train,x_test,y_train,y_test=train_test_split(x,y,test_size=0.3)

model_rf=RandomForestClassifier(n_estimators=20).fit(x_train,y_train)

score_rf=model_rf.score(x_test,y_test)

print(score_rf)

x_train,x_test,y_train, y_test=train_test_split(x,y,test_size=0.3)

model_svm=svm.SVC(kernel='rbf',gamma='auto',probability=True).fit(x_train,y_train)

score_svm=model_svm.score(x_test,y_test)

print(score_svm)

Cs=np.logspace(-1,3,10,base=2)

gammas=np.logspace(-4,1,50,base=2)

param_grid=dict(C=Cs,gamma=gammas)

grid=GridSearchCV(svm.SVC(kernel='rbf'),param_grid=param_grid,cv=10).fit(x,y)

print(grid.best_params_)

C=grid.best_params_['C']

gamma=grid.best_params_['gamma']

x_train, x_test,y_train,y_test=train_test_split(x,y,test_size=0.3)

model_svm=svm.SVC(kernel='rbf',C=C,gamma=gamma,probability=True).fit(x_train,y_train)

score_svm=model_svm.score(x_test,y_test)

print(score_svm)

rkf=RepeatedKFold(n_splits=3,n_repeats=2)

for train_index,test_index in rkf.split(x):

x_train=x.iloc[train_index]

x_test=x.iloc[test_index]

y_train=y.iloc[train_index]

y_test=y.iloc[test_index]

model_svm=svm.SVC(kernel='rbf',C=C,gamma=gamma,probability=True).fit(x_train,y_train)

score_svm=model_svm.score(x_test,y_test)

print(score_svm)

import matplotlib.pyplot as plt

import os

%matplotlib inline

x_values=np.arange(len(index))

print(x_values)

y_values=coef[coef!=0]

plt.bar(x_values,y_values

,color='lightblue'

,edgecolor='black'

,alpha=0.8

)

plt.xticks(x_values,index

,rotation='vertical'

,ha='right'

,va='top'

)

plt.xlabel('feature')

plt.ylabel('weight')

desktop_path=os.path.join(os.path.expanduser("C:/Users/Lenovo"),"Desktop")

output_file_path=os.path.join(desktop_path,"plot_output3.pdf")

plt.savefig(output_file_path,format='pdf')

from sklearn.metrics import roc_curve,roc_auc_score

y_probs=model_svm.predict_proba(x)

#print(y_probs)

#print (y_probs[:,1])

fpr,tpr,thresholds=roc_curve(y,y_probs[:,1],pos_label=1)

plt.plot(fpr,tpr,marker='o')

plt.xlabel("fpr")

plt.ylabel("tpr")

plt.show()

auc_score=roc_auc_score(y,model_svm.predict(x))

print(auc_score)

import matplotlib.pyplot as plt

from matplotlib.ticker import MultipleLocator

coefs=model_lassoCV.path(x,y,alphas=alphas,max_iter=100000)[1].T

non_zero_counts=np.sum(coefs!=0,axis=1)

MSEs=model_lassoCV.mse_path_

MSEs_mean=np.apply_along_axis(np.mean,1,MSEs)

MSEs_std=np.apply_along_axis(np.std,1,MSEs)

plt.figure()#dpi=300

plt.errorbar(model_lassoCV.alphas_,MSEs_mean

,yerr=MSEs_std

,fmt="o"

,ms=3

,mfc="r"

,mec="r"

,ecolor="lightblue"

,elinewidth=2

,capsize=4

,capthick=1)

plt.semilogx()

plt.axvline(model_lassoCV.alpha_,color='black',ls="--")

plt.xlabel('Lambda')

plt.ylabel('MSE')

num_lambdas=len(model_lassoCV.alphas_)

step=num_lambdas//10

selected_lambdas=model_lassoCV.alphas_[::step][:10]

selected_counts=non_zero_counts[::step][:10]

for alpha,count in zip(selected_lambdas, selected_counts):

plt.text(alpha,1.05*max(MSEs_mean+MSEs_std),str(count),ha='center',va='bottom',fontsize=8)

ax=plt.gca()

y_major_locator=MultipleLocator(0.05)

ax.yaxis.set_major_locator(y_major_locator)

desktop_path=os.path.join(os.path.expanduser("C:/Users/Lenovo"),"Desktop")

output_file_path=os.path.join(desktop_path,"plot_output2.pdf")

plt.savefig(output_file_path,format='pdf')

plt.show()

import os

coefs=model_lassoCV.path(x,y,alphas=alphas,max_iter=100000)[1].T

non_zero_counts=np.sum(coefs!=0, axis=1)

plt.figure()

plt.semilogx(model_lassoCV.alphas_,coefs,'-')

plt.axvline(model_lassoCV.alpha_,color='black',ls='--')

plt.xlabel('Lambda')

plt.ylabel('Coefficients')

num_lambdas=len(model_lassoCV.alphas_)

step=num_lambdas//10

selected_lambdas=model_lassoCV.alphas_[::step][:10]

selected_counts=non_zero_counts[::step][:10]

for alpha,count in zip(selected_lambdas, selected_counts):

plt.text(alpha,1.05*max(coefs.flatten()),str(count),ha='center',va='bottom',fontsize=8)

plt.ylim([min(coefs.flatten()),1.1*max(coefs.flatten())])#Ajust y-axis limits to fit the text

desktop_path=os.path.join(os.path.expanduser("C:/Users/Lenovo"),"Desktop")

output_file_path=os.path.join(desktop_path,"plot_output.pdf")

plt.savefig(output_file_path,format='pdf')

plt.show()

**Code for Developing and Validating Machine Learning Models**

import pandas as pd

import numpy as np

import matplotlib.pyplot as plt

from sklearn.model_selection import train_test_split, StratifiedKFold, RepeatedStratifiedKFold

from sklearn.linear_model import LogisticRegression

from sklearn.neighbors import KNeighborsClassifier

from xgboost import XGBClassifier

from sklearn.svm import SVC

from sklearn.ensemble import RandomForestClassifier

from sklearn.metrics import roc_curve, auc, accuracy_score, recall_score, confusion_matrix

from sklearn.calibration import calibration_curve, CalibratedClassifierCV

import tkinter as tk

from tkinter import filedialog

import warnings

import matplotlib as mpl

warnings.filterwarnings('ignore')

mpl.rcParams['figure.dpi'] = 300

from sklearn.metrics import precision_score, recall_score, f1_score

root = tk.Tk()

root.withdraw()

file_path = filedialog.askopenfilename(filetypes=[("Excel files", "*.xlsx")])

data = pd.read_excel(file_path)

X = data.drop(columns=['Group'])

y = data['Group']

X_train, X_test, y_train, y_test = train_test_split(X, y, test_size=0.2, random_state=42)

log_reg = LogisticRegression(max_iter=1000)

svm = SVC(probability=True)

random_forest = RandomForestClassifier()

xgb = XGBClassifier(use_label_encoder=False, eval_metric='logloss')

cv = RepeatedStratifiedKFold(n_splits=5, n_repeats=3, random_state=42)

models = {'Logit': log_reg, 'SVM': svm, 'RF': random_forest, 'XGBoost': xgb}

# Train and evaluate models

plt.figure(figsize=(6, 6))

for model_name, model in models.items():

print(f"Evaluating {model_name}:")

# Compute ROC curve and AUC

tprs = []

aucs = []

mean_fpr = np.linspace(0, 1, 100)

for train, val in cv.split(X_train, y_train):

model.fit(X_train.iloc[train], y_train.iloc[train])

y_pred_prob = model.predict_proba(X_train.iloc[val])[:, 1]

#if model_name == 'SVM':

# y_pred_prob = 1 - y_pred_prob

fpr, tpr, thresholds = roc_curve(y_train.iloc[val], y_pred_prob)

tprs.append(np.interp(mean_fpr, fpr, tpr))

tprs[-1][0] = 0.0

roc_auc = auc(fpr, tpr)

aucs.append(roc_auc)

# Plot ROC curve

mean_tpr = np.mean(tprs, axis=0)

mean_tpr[-1] = 1.0

mean_auc = auc(mean_fpr, mean_tpr)

std_auc = np.std(aucs)

plt.plot(mean_fpr, mean_tpr, label=f'{model_name} (AUC = {mean_auc:.3f})', linewidth=2)

# Set plot properties

plt.plot([0, 1], [0, 1], linestyle='--', lw=2, color='gray', label='Reference', alpha=.8)

plt.xlim([0.0, 1.0])

plt.ylim([0.0, 1.0])

plt.gca().set_aspect('equal', adjustable='box')

plt.xlabel('1-Specificity', fontsize=16, labelpad=10)

plt.ylabel('Sensitivity', fontsize=16, labelpad=10)

plt.title('ROC Curves', fontsize=18, pad=15)

plt.legend(loc='lower right', fontsize=16)

plt.tight_layout()

plt.xticks(np.arange(0, 1.1, 0.1), fontsize=14)

plt.yticks(np.arange(0, 1.1, 0.1), fontsize=14)

# Display and save the plot

plt.savefig('roc_curves.jpg', dpi=300)

plt.show()

metrics = {'Accuracy': accuracy_score, 'Sensitivity': recall_score, 'Precision': precision_score, 'F1 Score': f1_score}

for model_name, model in models.items():

model.fit(X_train, y_train)

y_pred = model.predict(X_test)

precision = precision_score(y_test, y_pred)

recall = recall_score(y_test, y_pred)

f1 = f1_score(y_test, y_pred)

print(f"\n{model_name} Model:")

print(f"Precision: {precision:.3f}")

print(f"Recall: {recall:.3f}")

print(f"F1 Score: {f1:.3f}")

plt.figure(figsize=(6, 6))

for model_name, model in models.items():

print(f"Evaluating {model_name}:")

# Compute ROC curve and AUC

tprs = []

aucs = []

mean_fpr = np.linspace(0, 1, 100)

for train, val in cv.split(X_train, y_train):

model.fit(X_train.iloc[train], y_train.iloc[train])

y_pred_prob = model.predict_proba(X_train.iloc[val])[:, 1]

#if model_name == 'SVM':

# y_pred_prob = 1 - y_pred_prob

fpr, tpr, thresholds = roc_curve(y_train.iloc[val], y_pred_prob)

tprs.append(np.interp(mean_fpr, fpr, tpr))

tprs[-1][0] = 0.0

roc_auc = auc(fpr, tpr)

aucs.append(roc_auc)

# Plot ROC curve

mean_tpr = np.mean(tprs, axis=0)

mean_tpr[-1] = 1.0

mean_auc = auc(mean_fpr, mean_tpr)

std_auc = np.std(aucs)

plt.plot(mean_fpr, mean_tpr, label=f'{model_name}', linewidth=2)

# Set plot properties

plt.plot([0, 1], [0, 1], linestyle='--', lw=2, color='gray', label='Reference', alpha=.8)

plt.xlim([0.0, 1.0])

plt.ylim([0.0, 1.0])

plt.gca().set_aspect('equal', adjustable='box')

plt.xlabel('1-Specificity', fontsize=16, labelpad=10)

plt.ylabel('Sensitivity', fontsize=16, labelpad=10)

plt.title('ROC Curves', fontsize=18, pad=15)

plt.legend(loc='lower right', fontsize=16)

plt.tight_layout()

plt.xticks(np.arange(0, 1.1, 0.1), fontsize=14)

plt.yticks(np.arange(0, 1.1, 0.1), fontsize=14)

# Display and save the plot

plt.savefig('roc_curves2.jpg', dpi=300)

plt.show()

metrics = {'Accuracy': accuracy_score, 'Sensitivity': recall_score}

for model_name, model in models.items():

print(f"Evaluating {model_name}:")

model.fit(X_train, y_train)

y_pred = model.predict(X_test)

for metric_name, metric in metrics.items():

score = metric(y_test, y_pred)

print(f"{metric_name}: {score:.3f}")

tn, fp, fn, tp = confusion_matrix(y_test, y_pred).ravel()

specificity = tn / (tn + fp)

print(f"Specificity: {specificity:.3f}")

# Compute ROC curve and AUC

model_prob = CalibratedClassifierCV(model, cv=5)

model_prob.fit(X_train, y_train)

y_pred_prob = model_prob.predict_proba(X_test)[:, 1]

fpr, tpr, thresholds = roc_curve(y_test, y_pred_prob)

auc_score = auc(fpr, tpr)

print(f"AUC: {auc_score:.3f}\n")

from sklearn.metrics import mean_squared_error

plt.figure(figsize=(6, 6))

brier_scores = {}

for model_name, model in models.items():

print(f"Evaluating {model_name} (Calibration Curve):")

# Calibration Curve calculations

prob_true, prob_pred = [], []

for train, val in cv.split(X_train, y_train):

model.fit(X_train.iloc[train], y_train.iloc[train])

y_pred_prob = model.predict_proba(X_train.iloc[val])[:, 1]

fraction_of_positives, mean_predicted_value = calibration_curve(y_train.iloc[val], y_pred_prob, n_bins=10)

prob_true.append(np.interp(np.linspace(0, 1, 10), mean_predicted_value, fraction_of_positives))

prob_pred.append(np.linspace(0, 1, 10))

# Plot Calibration Curve

mean_prob_true = np.mean(prob_true, axis=0)

mean_prob_pred = np.mean(prob_pred, axis=0)

plt.plot(mean_prob_pred, mean_prob_true, label=model_name, marker='o')

# Calculate Brier score

brier_score = mean_squared_error(mean_prob_true, mean_prob_pred)

brier_scores[model_name] = brier_score

print(f"Brier Score for {model_name}: {brier_score:.4f}")

# Calibration curve formatting

plt.plot([0, 1], [0, 1], linestyle='--', lw=2, color='gray', label='Reference', alpha=.8)

plt.xlim([0.0, 1.0])

plt.ylim([0.0, 1.05])

plt.xlabel('Mean Predicted Probability', fontsize=14,labelpad=20)

plt.ylabel('Fraction of Positives', fontsize=14,labelpad=20)

plt.title('Calibration Curves', fontsize=16, y=1.05)

plt.legend(loc='lower right')

plt.xticks(np.arange(0, 1.1, 0.1), fontsize=14)

plt.yticks(np.arange(0, 1.1, 0.1), fontsize=14)

plt.legend(loc='lower right', fontsize=14, prop={'size': 14})

plt.show()

import numpy as np

import pandas as pd

from sklearn.linear_model import LogisticRegression

from sklearn.svm import SVC

from sklearn.ensemble import RandomForestClassifier

from sklearn.model_selection import train_test_split

from sklearn.metrics import roc_curve, roc_auc_score, accuracy_score, recall_score, confusion_matrix

from sklearn.calibration import calibration_curve, CalibratedClassifierCV

import matplotlib.pyplot as plt

# Define the threshold probabilities for the DCA curve

thresh_probs = np.arange(0.1, 0.9, 0.1)

# Create an empty list to store the DCA scores for each model

dca_scores = []

# Define the cost-benefit matrix

# Here, we assume that a true positive (TP) result is worth 1, a false positive (FP) result is worth -2,

# a false negative (FN) result is worth -1, and a true negative (TN) result is worth 0.

costbenefit = np.array([[0, -2], [-1, 1]])

# Define the feature and target variables

data = pd.read_excel(file_path)

X = data.drop(columns=['Group'])

y = data['Group']

# Split the data into training and test sets

X_train, X_test, y_train, y_test = train_test_split(X, y, test_size=0.2, random_state=42)

# Loop over the models and compute the DCA scores

for model_name, model in models.items():

print(f"Evaluating {model_name}:")

# Fit the model and make predictions on the test set

model.fit(X_train, y_train)

y_pred_prob = model.predict_proba(X_test)[:, 1]

y_pred = (y_pred_prob >= 0.5).astype(int)

# Compute the net benefit for a range of threshold probabilities

net_benefit = []

for thresh in thresh_probs:

tn, fp, fn, tp = confusion_matrix(y_test, (y_pred_prob >= thresh).astype(int)).ravel()

net_benefit.append(np.sum(costbenefit * np.array([[fp, tp], [fn, tn]])) / len(y_test))

# Store the DCA scores for the model

dca_scores.append(net_benefit)

# Plot the DCA curve

plt.plot(thresh_probs, net_benefit, label=model_name)

plt.scatter(thresh_probs[np.argmax(net_benefit)], np.max(net_benefit), s=100, marker='o', color='black')

print(f"Optimal threshold for {model_name}: {thresh_probs[np.argmax(net_benefit)]:.2f}")

print(f"DCA score for {model_name}: {np.max(net_benefit):.3f}\n")

# Format the plot

plt.xlabel('Threshold Probability')

plt.ylabel('Net Benefit')

plt.title('Decision Curve Analysis')

plt.legend()

plt.show()

import numpy as np

import pandas as pd

from sklearn.linear_model import LogisticRegression

from sklearn.svm import SVC

from sklearn.ensemble import RandomForestClassifier

from sklearn.model_selection import train_test_split

from sklearn.metrics import confusion_matrix

import matplotlib.pyplot as plt

def calculate_net_benefit(thresh_group, y_pred_prob, y_label):

net_benefit = []

for thresh in thresh_group:

y_pred_label = y_pred_prob > thresh

tn, fp, fn, tp = confusion_matrix(y_label, y_pred_label).ravel()

n = len(y_label)

net_benefit.append(tp / n - fp / n * (thresh / (1 - thresh)))

return net_benefit

def plot_dca(ax, thresh_group, net_benefit_model, color, model_name):

# Plot the DCA curve

ax.plot(thresh_group, net_benefit_model, color=color, lw=2, label=model_name)

# Shade the area where the model has net benefit greater than treating all

max_nb_idx = np.argmax(net_benefit_model)

if net_benefit_model[max_nb_idx] > net_benefit_all[max_nb_idx]:

ax.fill_between(thresh_group, net_benefit_model, net_benefit_all, color=color, alpha=0.2)

# Configure the plot aesthetics

ax.set_xlim(0, 1)

ax.set_ylim(-0.2, max(net_benefit_model) + 0.1)

ax.set_xlabel('Threshold Probability', fontsize=22, fontweight='normal', labelpad=20)

ax.set_ylabel('Net Benefit', fontsize=22, fontweight='normal', labelpad=20)

ax.set_title('Decision Curve Analysis', fontsize=24, fontweight='normal', y=1.05)

ax.grid(True, alpha=0.5)

ax.legend(loc='upper right', fontsize=24)

return ax

# Define the feature and target variables

data = pd.read_excel(file_path)

X = data.drop(columns=['Group'])

y = data['Group']

# Split the data into training and test sets

X_train, X_test, y_train, y_test = train_test_split(X, y, test_size=0.2, random_state=42)

# Define the threshold probabilities for the DCA curve

thresh_group = np.arange(0, 1.01, 0.01)

# Calculate the net benefit for treating all patients

net_benefit_all = np.zeros_like(thresh_group)

tn, fp, fn, tp = confusion_matrix(y_test, np.ones_like(y_test)).ravel()

n = len(y_test)

net_benefit_all = tp / n - fp / n * (thresh_group / (1 - thresh_group))

# Loop over the models and compute the DCA curve

fig, ax = plt.subplots(figsize=(12, 10))

# Plot the Treat All and Treat None lines before the loop

ax.plot(thresh_group, net_benefit_all, color='gray', lw=2, linestyle='--', label='Treat All')

ax.plot([0, 1], [0, 0], color='gray', lw=2, linestyle=':', label='Treat None')

colors = [ 'blue','orange','green','red','purple',]

for i, (model_name, model) in enumerate(models.items()):

print(f"Evaluating {model_name}:")

# Fit the model and make predictions on the test set

model.fit(X_train, y_train)

y_pred_prob = model.predict_proba(X_test)[:, 1]

# Calculate the net benefit for the model on the test set

net_benefit_model = calculate_net_benefit(thresh_group, y_pred_prob, y_test)

# Plot the DCA curve with a different color for each model

ax = plot_dca(ax, thresh_group, net_benefit_model, color=colors[i], model_name=model_name)

print(f"Optimal threshold for {model_name}: {thresh_group[np.argmax(net_benefit_model)]:.2f}")

print(f"DCA score for {model_name}: {np.max(net_benefit_model):.3f}\n")

plt.xticks(np.arange(0, 1.1, 0.1), fontsize=20)

plt.yticks(np.arange(0, 1.1, 0.1), fontsize=20)

plt.show()

# Fit the model and make predictions on the test

fig.savefig("plotDCA1.jpg", dpi=300, bbox_inches='tight')

import pandas as pd

import numpy as np

from sklearn import svm

from sklearn.model_selection import train_test_split

from sklearn.preprocessing import StandardScaler

import shap

import matplotlib.pyplot as plt

import warnings

import tkinter as tk

from tkinter import filedialog

from PIL import Image

warnings.filterwarnings('ignore')

shap.initjs()

import matplotlib as mpl

mpl.rcParams['figure.dpi'] = 300

##models = {'Logit': log_reg, 'SVM': svm, 'RF': random_forest, 'XGBoost': xgb}

model2 = svm

explainer = shap.Explainer(model2, X_train, feature_names=data.columns[1:])

shap_values = explainer(X_test)

shap.plots.force(shap_values)

shap.plots.beeswarm(shap_values)

plt.savefig("beeswarm_plot.jpg", dpi=300, format='jpg')

plt.close()

shap.plots.bar(shap_values)

plt.savefig("bar_plot.jpg", dpi=300, format='jpg')

plt.close()

mean_shap_values = np.mean(np.abs(shap_values.values), axis=0)

max_contrib_index = np.argmax(mean_shap_values)

min_contrib_index = np.argmin(mean_shap_values)

max_contrib_feature = shap_values.feature_names[max_contrib_index]

min_contrib_feature = shap_values.feature_names[min_contrib_index]

shap.plots.force(shap_values[:, max_contrib_index])

shap.plots.force(shap_values[:, min_contrib_index])

root = tk.Tk()

root.withdraw()

file_path = filedialog.askopenfilename(filetypes=[("Excel files", "*.xlsx")])

data2 = pd.read_excel(file_path)

X_validation = data2.drop(columns=['Group'])

y_validation = data2['Group']

y_pred_prob = svm.predict_proba(X_validation)[:, 1]

#y_pred_prob = log_reg.predict_proba(X_validation)[:, 1]

#y_pred_prob = random_forest.predict_proba(X_validation)[:, 1]

fpr, tpr, thresholds = roc_curve(y_validation, y_pred_prob)

roc_auc = auc(fpr, tpr)

plt.figure(figsize=(6, 6))

plt.plot(fpr, tpr, label=f'SVM', linewidth=2)

plt.xlabel('False Positive Rate')

plt.ylabel('True Positive Rate')

plt.legend(loc='lower right')

# Set plot properties

plt.plot([0, 1], [0, 1], linestyle='--', lw=2, color='gray', label='Reference', alpha=.8)

plt.xlim([0.0, 1.0])

plt.ylim([0.0, 1.0])

plt.gca().set_aspect('equal', adjustable='box')

plt.xlabel('1-Specificity', fontsize=16, labelpad=10)

plt.ylabel('Sensitivity', fontsize=16, labelpad=10)

plt.title('ROC Curves', fontsize=18, pad=15)

plt.legend(loc='lower right', fontsize=16)

plt.tight_layout()

plt.xticks(np.arange(0, 1.1, 0.1), fontsize=14)

plt.yticks(np.arange(0, 1.1, 0.1), fontsize=14)

# Display and save the plot

plt.savefig('roc_curves_validtion.jpg', dpi=300)

plt.show()

metrics = {'Accuracy': accuracy_score, 'Sensitivity': recall_score}

roc_auc

prob_true, prob_pred = calibration_curve(y_validation, y_pred_prob, n_bins=10)

plt.figure(figsize=(6, 6))

plt.plot(prob_pred, prob_true, label='SVM', marker='o')

# Calibration curve formatting

plt.plot([0, 1], [0, 1], linestyle='--', lw=2, color='gray', label='Reference', alpha=.8)

plt.xlabel('Mean Predicted Probability', fontsize=14,labelpad=20)

plt.ylabel('Fraction of Positives', fontsize=14,labelpad=20)

plt.title('Calibration Curves', fontsize=16, y=1.05)

plt.legend(loc='lower right')

plt.xticks(np.arange(0, 1.1, 0.1), fontsize=14)

plt.yticks(np.arange(0, 1.1, 0.1), fontsize=14)

plt.legend(loc='lower right', fontsize=14, prop={'size': 14})

# Save the figure

plt.savefig('calibration_curve.jpg', dpi=300, bbox_inches='tight')

plt.show()

def calculate_net_benefit(thresh_group, y_pred_prob, y_label):

net_benefit = []

for thresh in thresh_group:

y_pred_label = y_pred_prob > thresh

tn, fp, fn, tp = confusion_matrix(y_label, y_pred_label).ravel()

n = len(y_label)

net_benefit.append(tp / n - fp / n * (thresh / (1 - thresh)))

return net_benefit

def plot_dca(ax, thresh_group, net_benefit_model, color, model_name):

# Plot the DCA curve

ax.plot(thresh_group, net_benefit_model, color=color, lw=2, label=model_name)

# Shade the area where the model has net benefit greater than treating all

max_nb_idx = np.argmax(net_benefit_model)

if net_benefit_model[max_nb_idx] > net_benefit_all[max_nb_idx]:

ax.fill_between(thresh_group, net_benefit_model, net_benefit_all, color=color, alpha=0.2)

# Configure the plot aesthetics

ax.set_xlim(0, 1)

ax.set_ylim(-0.2, max(net_benefit_model) + 0.1)

ax.set_xlabel('Threshold Probability', fontsize=22, fontweight='normal', labelpad=20)

ax.set_ylabel('Net Benefit', fontsize=22, fontweight='normal', labelpad=20)

ax.set_title('Decision Curve Analysis', fontsize=24, fontweight='normal', y=1.05)

ax.grid(True, alpha=0.5)

ax.legend(loc='upper right', fontsize=24)

return ax

# Define the threshold probabilities for the DCA curve

thresh_group = np.arange(0, 1.01, 0.01)

# Calculate the net benefit for treating all patients

net_benefit_all = np.zeros_like(thresh_group)

tn, fp, fn, tp = confusion_matrix(y_validation, np.ones_like(y_validation)).ravel()

n = len(y_validation)

net_benefit_all = tp / n - fp / n * (thresh_group / (1 - thresh_group))

# Compute DCA curve

fig, ax = plt.subplots(figsize=(12, 10))

# Plot the Treat All and Treat None lines before the loop

ax.plot(thresh_group, net_benefit_all, color='gray', lw=2, linestyle='--', label='Treat All')

ax.plot([0, 1], [0, 0], color='gray', lw=2, linestyle=':', label='Treat None')

# Calculate the net benefit for the model on the validation set

net_benefit_model = calculate_net_benefit(thresh_group, y_pred_prob, y_validation)

# Plot the DCA curve with a different color for each model

ax = plot_dca(ax, thresh_group, net_benefit_model, color='red', model_name='SVM')

print(f"Optimal threshold for XGB: {thresh_group[np.argmax(net_benefit_model)]:.2f}")

print(f"DCA score for XGB: {np.max(net_benefit_model):.3f}\n")

plt.xticks(np.arange(0, 1.1, 0.1), fontsize=20)

plt.yticks(np.arange(0, 1.1, 0.1), fontsize=20)

plt.show()

fig.savefig("plotDCA_validation.jpg", dpi=300, bbox_inches='tight')
